# Patient and Physician Perspectives on the Use of a Connected Ecosystem for Diabetes Management: International Cross-Sectional Observational Study

**DOI:** 10.2196/47145

**Published:** 2023-11-30

**Authors:** Elizabeth Benito-Garcia, Julio Vega, Eric J Daza, Wei-Nchih Lee, Adee Kennedy, Jean-Marc Chantelot

**Affiliations:** 1 Sanofi Paris France; 2 Evidation Health, Inc San Mateo, CA United States; 3 Sanofi Bridgewater Township, NJ United States

**Keywords:** type 2 diabetes mellitus, insulin treatment, connected ecosystems, surveys, diabetes, diabetic, ecosystem, ecosystems, telehealth, telemedicine, eHealth, digital health, health technology, adoption, perception, attitude, intention, acceptance

## Abstract

**Background:**

Collaboration between people with type 2 diabetes (T2DM) and their health care teams is important for optimal control of the disease and outcomes. Digital technologies could potentially tie together several health care-related devices and platforms into connected ecosystems (CES), but attitudes about CES are unknown.

**Objective:**

We surveyed convenience samples of patients and physicians to better understand which patient characteristics are associated with higher likelihoods of (1) participating in a potential CES program, as self-reported by patients with T2DM and (2) clinical benefit from participation in a potential CES program, as reported by physicians.

**Methods:**

Adults self-reporting a diagnosis of T2DM and current insulin use (n=197), and 33 physicians whose practices included ≥20% of such patients, were enrolled in the United States, France, and Germany. We surveyed both groups about the likelihood of patient participation in a CES. We then examined the associations between patients’ clinical and sociodemographic characteristics and this likelihood. We also described characteristics of patients likely to clinically benefit from CES use, according to physicians.

**Results:**

Compared with patients in Germany and France, US patients were younger (mean age 45.3 [SD 11.9] years vs 61.9 [SD 9.2] and 65.8 [SD 9.4] years, respectively), more often female, more highly educated, and more often working full-time. In all, 51 (44.7%) US patients, 16 (36.4%) German patients, and 18 (46.3%) French patients indicated strong interest in a CES program, and 115 (78.7%) reported currently using ≥1 connected device or app. However, physicians believed that only 11.3%-19.2% of their patients were using connected devices or apps to manage their disease. Physicians also reported infrequently recommending or prescribing connected devices to their patients, although ≥80% (n=28) of them thought that a CES could help support their patients in managing their disease. The factors most predictive of patient likelihood of participating in a CES program were cost, inclusion of medication reminders, and linking blood glucose levels to behaviors such as eating and exercise. In all countries, the most common patient expectations for a CES program were that it could help them eat more healthfully, increase their physical activity, increase their understanding of how blood glucose relates to behavior such as exercise and eating, and reduce stress. Physicians thought that newly diagnosed patients, sicker patients—those who had been hospitalized for diabetes, were currently using insulin, or who had any comorbid condition—and patients who were nonadherent to treatment were most likely to benefit from CES use.

**Conclusions:**

In this study, there was a high degree of interest in the future use of CES, although additional education is needed among both patients with T2DM and their physicians to achieve the full potential of such systems to improve self-management and clinical care for the disease.

## Introduction

Type 2 diabetes mellitus (T2DM) affects more than 400 million people worldwide, and its prevalence is rapidly increasing [1]. In the United States alone, the total cost of diagnosed diabetes was estimated at US $327 billion in 2017, with 1 of every 4 health care dollars spent on caring for people with the disease [2]. Diabetes has been associated with diminished physical, mental, psychological, and social quality of life [3] and is a leading cause of morbidity and death [4]. Given these immense personal and societal costs, more work is needed to better understand how to support patients and their health care teams in diabetes management.

The American Diabetes Association identifies glycemic control as an “integral component of effective therapy of patients taking insulin.” They recommend a collaborative approach between individuals with diabetes and physician-coordinated medical teams to provide continuing education and evaluations of patient technique in the use and results of glucose monitoring technologies to help patients achieve glycemic control [5]. However, only 48% of US adults with diabetes received educational support in one large surveillance study, and only 61.5% of the adults in that study monitored their blood glucose at least daily [6]. Similarly, only 10%-20% of patients in a large longitudinal study in France complied with guideline recommendations for medical monitoring of their disease [7], and only 42% of patients in Germany were participating in a diabetes management program [8].

As innovative digital health tools, platforms, and therapeutics continue to be developed and released across the world, people with diabetes are increasingly exposed to technologies that aim to help them better manage their disease. As one example, the use of a mobile smartphone app that included Bluetooth-enabled glucose monitoring, in-app education and support, and activity tracker data was associated with lower hemoglobin A_1c_ levels at 3 months in patients with type 1 diabetes compared with use of the app or monitoring or education without activity tracker data [9]. Combining networks of tools and monitoring capabilities into a single, holistic connected ecosystem (CES) could provide increased support to both patients who require improved glycemic control and their physicians.

The optimal diabetes CES would be a comprehensive, integrated, end-to-end global solution that simplifies diabetes management, allowing direct collaborations between patients and their physicians. Such a program could deliver personalized recommendations to help patients better manage their disease. For physicians, such programs could identify patients at higher risk for worse disease outcomes. The use of a CES thus might improve outcomes and cost-effectiveness, reduce financial risk for payers, and improve decision-making, practice efficiency, and effectiveness of care for health care practitioners.

The goals of this study were to better understand the patient characteristics (eg, sociodemographic, clinical, and behavioral) that are associated with higher likelihoods of (1) participating in a potential CES program, as self-reported by patients with diabetes and (2) clinically benefiting from such participation, as reported by physicians, in 3 countries with modern health care delivery systems. The insights from this study can inform the degree to which patients and physicians use digital devices in the management of T2DM and the limitations that must be addressed to optimize both future studies of these systems and their possible benefits to patients and physicians.

## Methods

### Study Design

This cross-sectional observational study consisted of a survey on a convenience sample of patients with T2DM in the United States, France, and Germany and physicians caring for patients with T2DM in the same countries. The study was decentralized: a dedicated web-based platform was used to identify, advertise, recruit, verify eligibility, consent, and enroll participants.

### Ethical Considerations

In the United States, the Solutions institutional review board (Yarnell, Arizona) reviewed and approved the study protocol (approval number 2021/11/25). In France and Germany, the ethics committees did not consider the study to represent human subject research; therefore, no approval was necessary.

### Participants

Eligible patients were at least aged 18 years; lived in one of the 3 countries; spoke, read, and understood the local language (ie, English, French, or German); self-reported a diagnosis of T2DM; had received basal insulin or multidrug injection treatment for diabetes within the previous 12 months; and were currently being treated with insulin. Potential patient participants were excluded for self-reported type 1 diabetes mellitus or significant cognitive impairment or dementia. We limited eligibility to insulin-dependent patients because its use can be a marker for more severe diabetes [10]; the use of a CES might thus offer such patients greater benefit.

Eligible physicians were also at least aged 18 years; lived in 1 of the 3 countries; and spoke, read, and understood the local language. They were also required to be licensed and practicing medicine in the country of interest and to have ≥20% of their patients receiving insulin within the past 12 months.

In the United States, all patient and physician participants were members of the free mobile Achievement health and research platform (Evidation Health, Inc), which represents more than 4 million individuals spanning all 50 states and 90% of zip codes [11]. The platform delivers personalized tools and insights to motivate and empower people to manage their health. Members can connect fitness apps, activity trackers, and other health-related tools to the platform and share self-reported medical information. Achievement relies solely on member-generated records; it cannot access clinical or claims data.

In France and Germany, the pool of potential T2DM participants was derived from the Carenity platform, an online patient community for people with chronic diseases [12]. Physicians from France and Germany were recruited from a Carenity partner vendor that specializes in physician surveys. Eligible physician respondents were directed to the Carenity platform, where the web-based surveys were conducted.

### Recruitment, Screening, and Enrollment

Potential patient participants in each country were those who had self-reported a diagnosis of T2DM, their insulin use, exposure to diabetes management apps, and duration of insulin treatment. Potential physician participants in each country were those who had self-reported being a medical doctor or doctor of osteopathy. Throughout recruitment, we monitored the distributions of patient sex and duration of insulin treatment to achieve targets of 50% female, 50% male, ≥60% receiving insulin for ≥6 months, and ≥20% receiving insulin for <6 months, respectively.

Evidation Health, Inc sent potential US participants a link to the landing page on the Achievement mobile platform, where they were presented with an overview of the study. Those who expressed interest were then directed to complete a web-based screening survey, which collected demographic data and either medical history, comorbidities, and risk factors (patients) or professional and practice characteristics (physicians). Respondents were deemed eligible for enrollment if they met all inclusion criteria and none of the exclusion criteria. Potential participants who did not complete the screening steps were reminded and contacted by email, text message, push notification, or phone call, or a combination of these methods.

In Germany and France, adults with self-reported T2DM on the Carenity platform were informed by email of the purpose of the survey and offered to participate. Similar to the United States, distributions of sex and time on insulin were monitored for French and German participants recruited by Carenity to ensure the target sex breakdown (50% male and 50% female) and time on insulin breakdown (targeting ≥60% who had been on insulin for ≥6 months and ≥20% who had been receiving insulin for <6 months) were being achieved. Physician panels from the Carenity vendor were sent invitations to participate in the survey, and interested physicians were sent instructions to use the Carenity platform for eligibility screening and survey completion.

### Study Procedures

After eligibility was verified, signed informed consent was obtained from participants prior to enrollment using Evidation Health’s eConsent process (United States) or via the Carenity platform (Germany and France). They were then asked to complete the 1-time web-based survey (see Multimedia Appendix 1 for the complete survey instruments for patients and physicians). Further, in addition to the variables collected during screening, the survey asked patients a total of 47 questions on 9 screens about their socioeconomic background, health care usage, diabetes management (including the past and current use of devices for this purpose), usefulness of such devices for disease management (score of ≥7 on a scale of 0 to 10), and interest in and likelihood of engaging in a hypothetical CES program on a scale of 0 to 10 (the United States) or 0 to 1000 (Germany and France). The question was phrased as “A Connected EcoSystem (CES) is a tool that you could use to improve your Diabetes management. It would be designed to help you keep your blood sugar under control. It could include using a digital device to track your insulin delivery and helping you monitor your blood sugar (glucose) levels. You could also talk with your health care provider about your treatment with the CES tool.” They were also asked about whether various factors would be very important to them in a CES program, defined as a score of 10 on a 0-10 scale. Patient diabetes-related goals and degree of assistance desired in achieving these goals were also assessed.

The web-based survey asked physicians a total of 33 questions on 6 screens about their demographic, professional, and practice characteristics as well as their patients’ characteristics, health care usage, device usage for diabetes management, and characteristics of patients who would derive medical benefit from a CES program. The survey also assessed the physicians’ attitudes toward, potential interest in, and likelihood of recommending or prescribing a CES program, which was described as “A Connected Ecosystem (CES) is a network of tools and capabilities to support patients requiring improved glycemic control. For a patient, this may mean using a digital device to track insulin delivery or to monitor his/her blood glucose levels, or provide a digital support platform to discuss with his/her health care provider on appropriate therapy. For physicians, a CES can help improve decision making, practice efficiency, and provide effective care for patients. The goal of the CES is to help identify patients at higher risk for worse disease outcomes and improve disease management.” Attitudes were assessed by a 5-category scale of strongly disagree, disagree, neutral, agree, and strongly agree in response to various statements. The likelihood of recommending or prescribing a CES program was assessed as very unlikely, somewhat unlikely, neutral, somewhat likely, or very likely.

Participants were considered enrolled in the study after completing the web-based survey, which was available for about 14 days after the participant signed the informed consent form. If participants provided consent but did not complete the survey, they were reminded by means of email, text message, push notification, or phone calls.

Participants (patients and physicians) in the United States received US $20 for completing the survey, in the form they selected (eg, bank transfer, physical debit card, or electronic debit card). The compensation amounts were described to participants during the informed consent process. Patient and physician participants in France and Germany were compensated at the same level as US participants but in their local currencies.

### Data Security

Evidation Health, Inc secured all identifiable information about participants from the United States. Data were transmitted via secure encrypted protocols and stored on encrypted disks on secure and hardened servers, access to which was limited to necessary information technology staff at Evidation Health, Inc. Personally identifiable information was accessible only by a restricted set of persons within Evidation Health, Inc, and was used only for distributing study material and for participant support. Researchers at Evidation Health, Inc had access only to deidentified study data. Personally identifiable information for patients in Germany and France was secured by Carenity and removed before delivery to Evidation Health, Inc.

### Sample Size Calculation

No formal sample size calculation was performed for this descriptive observational study of convenience samples.

### Statistical Analysis

We excluded patients from the analysis if they had unreliable data from the Achievement platform (United States only) or on the full web-based survey. These could include improbable parenthood age, weight, or sleep time, straight-lined answers, or having a completion time that was considered “too short” (the top 5% of completion times). We excluded physicians from the analysis if they had unreliable data from the Achievement platform (United States only) or on the full web-based survey, to include a completion time of <5 minutes (as per consensus between the analyst and principal investigator), an unrealistic patient load, or unlikely presence of comorbidities (eg, obesity and stroke) based on the community’s prevalence.

For patient participants, we generated summary statistics for clinical and sociodemographic characteristics, overall and stratified by the first primary end point: self-reported likelihood of future CES use. For physician participants, we likewise created summary statistics describing their patient populations, and we examined the associations between patient characteristics and the physician-estimated likelihood of the patients benefiting medically from a CES, the second primary end point. Safety data were not routinely collected in this observational survey study.

Continuous variables were summarized as means with SDs and minimums and maximums, or as medians with IQRs. Categorical variables were summarized as frequencies with percentages. β coefficients (and their respective 95% CIs) were calculated to identify which patient characteristics were associated with each outcome. All statistical hypothesis tests were conducted with an overall maximum tolerable false positive rate (ie, chance of a false finding) of >.05. When needed, this rate was adjusted to account for multiple testing or multiplicity using a correction for the false discovery rate.

## Results

### Patients

From January 27, 2022, to April 28, 2022, a total of 261 patients were enrolled: 161 in the United States and 50 each in Germany and France. Of these, a total of 64 patients were excluded from the analysis, leaving 197 patients: 114 in the United States, 44 in Germany, and 39 in France. The demographic and clinical characteristics of these participants are shown in Table 1.

Compared with patients in Germany and France, US patients were considerably younger, more often female, more highly educated, and much more likely to be working full-time. They were also more likely to have started insulin treatment within the previous 6 months and least likely to be using short-acting insulin. German patients were most likely to be using insulin multiple times per day, and French patients, who had the lowest BMI value, were least likely to report the use of diet and exercise to control their diabetes. German patients were also much more likely to report that their diabetes was well controlled, but they reported the highest proportions having cardiovascular disease and heart failure. In contrast, French patients had the highest proportions reporting an elevated hemoglobin A_1c_ level and renal disease.

**Table 1:** Self-reported demographic and clinical characteristics of the patients surveyed.

Characteristics		United States (n=114)	Germany (n=44)	France (n=39)
**Age (years)**				
	Mean (SD)	45.3 (11.9)	61.9 (9.2)	65.8 (9.4)
	Minimum-maximum	19-71	35-79	41-81
**Sex, n (%)**				
	Female	76 (66.7)	17 (38.6)	16 (41.0)
	Male	38 (33.3)	27 (61.4)	23 (59.0)
**BMI (kg/m^2^), mean (SD)**		34.8 (9.3)	35.4 (8.8)	31.7 (6.2)
	Minimum-maximum	20.3-68.1	21.5-67.1	18.2-49.2
Bachelor’s degree or higher, n (%)		55 (48.2)	8 (18.2)	7 (17.9)
**Employment, n (%)**				
	Full-time	79 (69.3)	5 (11.4)	4 (10.3)
	Part-time	10 (8.8)	5 (11.4)	4 (10.3)
	Unemployed or retired	8 (7.0)	26 (59.1)	28 (71.8)
Have a primary care physician, n (%)		106 (93)	44 (100)	39 (100)
Insulin use <6 months, n (%)		15 (13.2)	2 (4.6)	2 (5.2)
**Any use of insulin, n (%)**				
	Long-acting analog	81 (71.1)	35 (79.5)	23 (59.0)
	Short-acting	46 (40.4)	23 (52.3)	17 (43.6)
	Multidrug injection	30 (26.3)	15 (34.1)	6 (15.4)
**Current use of insulin, n (%)**				
	Long-acting analog, alone, or in combination	73 (64.0)	31 (70.5)	22 (56.4)
	Short-acting, alone, or in combination	31 (27.2)	20 (45.5)	18 (46.2)
**Frequency of insulin use, n (%)**				
	Once per day	60 (52.6)	12 (27.3)	16 (41.0)
	Multiple times per day	35 (30.7)	25 (56.8)	12 (30.8)
**Noninsulin diabetes management methods, n (%)**				
	Oral medications	92 (80.7)	38 (86.4)	32 (82.1)
	Diet and exercise	70 (61.4)	23 (52.3)	13 (33.3)
	Oral medications, diet, and exercise	41 (36.0)	15 (34.1)	10 (25.6)
Diabetes was well controlled, n (%)		68 (59.6)	33 (75.0)	22 (56.4)
Hemoglobin A_1c_ >7.5%, n (%)		30 (26.3)	8 (18.2)	14 (35.9)
**Diabetes-related complications, n (%)**				
	Cardiovascular disease	5 (4.4)	9 (20.5)	6 (15.4)
	Heart failure	1 (0.9)	6 (13.6)	2 (5.1)
	Renal disease	3 (2.6)	1 (2.3)	5 (12.8)
	Stroke	0 (0)	1 (2.3)	2 (5.1)

#### Likelihood of Participating in a CES Program

Patients in all 3 countries expressed strong interest in participating in a CES program. In all, 51 (44.7%) US patients, 16 (36.4%) German patients, and 18 (46.2%) French patients reported a score of 9 or 10 for the likelihood they would participate in such a program. The median scores for this question were 8.0/10 (IQR 6.0-10) for the United States, 740.0/1000 (497.5-965.0) for Germany, and 814.0/1000 (519.5-1000) for France.

**Figure 1:**
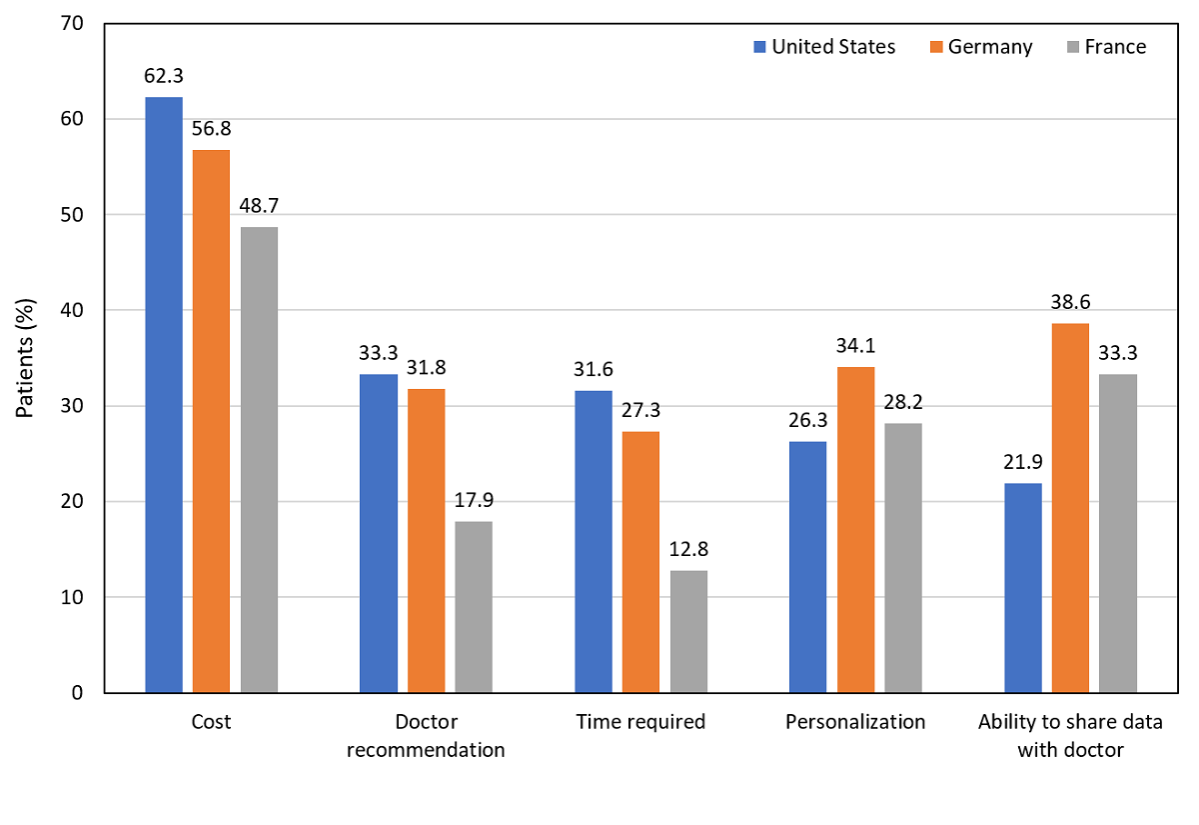
CES factors considered very important to patients surveyed. CES: connected ecosystem.

Cost was the factor considered most important in a CES in all 3 countries (Figure 1). The time required for use was least important to French patients, as was a doctor’s recommendation for a CES. The ability to share data with their doctors was less important to US patients compared with German and French patients.

#### Use of Connected Devices

The use of connected devices was common, and they were generally considered useful (Table 2; Figures S1-S3 in Multimedia Appendix 2). The most common devices used among the 3 countries at the time of the survey were smartwatches, flash or continuous blood glucose monitors, and smart blood pressure monitors. The proportion of patients who considered these devices useful ranged from 38.8% in France (for blood pressure monitors; n=7) to 96.1% in the United States (for glucose monitors; n=25).

**Table 2:** Any and current use of connected devices by patients.

Devices, n (%)	United States (n=114)		Germany (n=44)		France (n=39)	
	Any use	Current use	Any use	Current use	Any use	Current use
Smartwatch	59 (51.8)	41 (36)	20 (45.5)	10 (22.7)	13 (33.3)	0 (0)
Glucose monitor	26 (22.8)	20 (17.5)	31 (70.5)	16 (36.4)	27 (69.2)	16 (41)
Smart BP monitor	—^a^	—	25 (56.8)	16 (36.4)	18 (46.2)	3 (7.7)
Smart scale	—	—	13 (29.5)	4 (9.1)	12 (30.8)	3 (7.7)
None	37 (32.5)	19 (16.7)	5 (11.4)	9 (20.5)	8 (20.5)	14 (35.9)

^a^—: not available.

#### Patient Diabetes-Related Goals and Desire for Help

When asked about their goals, the patients indicated that they wanted to eat more healthfully, increase their physical activity, increase their understanding of how blood glucose relates to behaviors such as eating or other activities, and reduce stress (Table 3; Figures S4-S6 in Multimedia Appendix 2). German participants also reported concerns about unhealthy behaviors such as alcohol use and smoking as well as medication noncompliance. A minority of German patients—11.4% (n=5)—reported that they had no goals. Patients desired help with all of the goals to various degrees.

**Table 3:** Goals of patients with diabetes and desire for help with them.

Goals, n (%)	United States (n=114)		Germany (n=44)		France (n=39)	
	Goal	Desire help	Goal	Desire help	Goal	Desire help
Eat more healthfully	98 (86)	68 (59.6)	29 (65.9)	22 (50)	17 (43.6)	8 (20.5)
Increase physical activity	90 (78.9)	48 (42.1)	18 (40.9)	20 (45.5)	28 (71.8)	17 (43.6)
Better understand link between blood glucose and behavior	59 (51.8)	33 (28.9)	16 (36.4)	7 (15.9)	16 (41)	12 (30.8)
Reduce stress	56 (49.1)	33 (28.9)	19 (43.2)	9 (20.5)	10 (25.6)	8 (20.5)
Improve medication compliance	—^a^	—	19 (43.2)	4 (9.1)	—	—

^a^—: not available.

#### Expectations for a CES Program

In all 3 countries, the most common patient expectations for a CES program were that it could help them eat more healthfully, increase their physical activity, and increase their understanding of how blood glucose relates to behavior (Figures S7-S9 in Multimedia Appendix 2). About 1 in 4 US patients, along with about 1 in 3 patients in Germany and France, expected that a CES could also help them reduce stress.

The factor that patients in the US and France most wanted in a digital program to manage their diabetes was the ability to track blood glucose. Further, also mentioned were the ability to track medication usage and to talk to their doctor about their disease (United States), to track health-related information (eg, sleep and stress) and talk to their doctor about their disease (Germany), and to track other health-related information and to obtain general information about diabetes management (France).

#### Predictors of Willingness to Use a CES

The patient-generated characteristics most predictive of willingness to participate in a CES program in the United States were cost, inclusion of medication reminders, and the ability to track other health-related information (Figure S10 in Multimedia Appendix 2). For German patients, these were increasing their understanding of the link between blood glucose and behavior (positive association) and any reported use of intermediate-acting insulin (negative association; Figure S11 in Multimedia Appendix 2). The likelihood of CES participation among French patients tended to increase for obese participants and decrease if they reported measuring blood glucose levels once per week (Figure S12 in Multimedia Appendix 2).

### Physicians

From January 27, 2022, to April 28, 2022, a total of 65 physicians were enrolled: 45 in the United States and 10 each in Germany and France. Of these, 32 were excluded from the analysis because of unreliability of the data (eg, <5-minute survey completion time or unrealistic self-reported patient load), leaving 33 physicians: 15 in the United States, 10 in Germany, and 8 in France. Most physicians in the United States were female, were working in internal medicine or other specialties, and had been practicing for ≤10 years (Table 4). In contrast, the physicians in the other 2 countries were predominantly male, were working in endocrinology or family medicine, and had been practicing for more than 10 years.

**Table 4:** Demographic and professional characteristics of the physicians surveyed.

Characteristics, n (%)		United States (n=15)	Germany (n=10)	France (n=8)
**Sex**				
	Female	8 (53.3)	3 (30)	0 (0)
	Male	7 (46.7)	7 (70)	8 (100)
**Medical specialty**				
	Endocrinology	1 (6.7)	5 (50)	1 (12.5)
	Internal medicine	5 (33.3)	3 (30)	—
	Family medicine	3 (20)	2 (20)	7 (87.5)
	Other	6 (40)	—^a^	—
**Years practicing medicine**				
	0-10	8 (53.3)	—	—
	11-20	4 (26.7)	7 (70)	1 (12.5)
	21-30	3 (20)	2 (20)	4 (50)
	>30	—	1 (10)	3 (37.5)

^a^—: not available.

#### Patient Characteristics

Physicians reported a wide distribution in the number of patients seen with diabetes (Table 5). US clinicians reported the lowest proportion of patients with glycemic control (mean 52%) and the highest proportion of patients having trouble adhering to their medications (mean 33.7%). French physicians gave the highest estimate for patients with glycemic control, whereas German physicians reported the lowest proportion of patients with medication nonadherence.

**Table 5:** Physician-reported characteristics of their patients with diabetes.

Characteristics		United States (n=15)	Germany (n=10)	France (n=8)
**Number of patients seen in the last year**				
	Mean (SD)	436.0 (305.6)	650.0 (541.1)	279.4 (152.0)
	Minimum-maximum	50.0-1000.0	150.0-2000.0	100.0-500.0
**Patients with glycemic control**				
	Mean (SD)	52.0 (20.0)	64.5 (26.9)	69.4 (16.8)
	Minimum-maximum	20.0-80.0	20.0-90.0	40.0-100.0
**Patients with trouble adhering to medications**				
	Mean (SD)	33.7 (24.1)	13.5 (5.3)	22.6 (12.1)
	Minimum-maximum	10.0-85.0	5.0-20.0	8.0-40.0

#### Physician Perceptions of Connected Device Use

**Figure 2:**
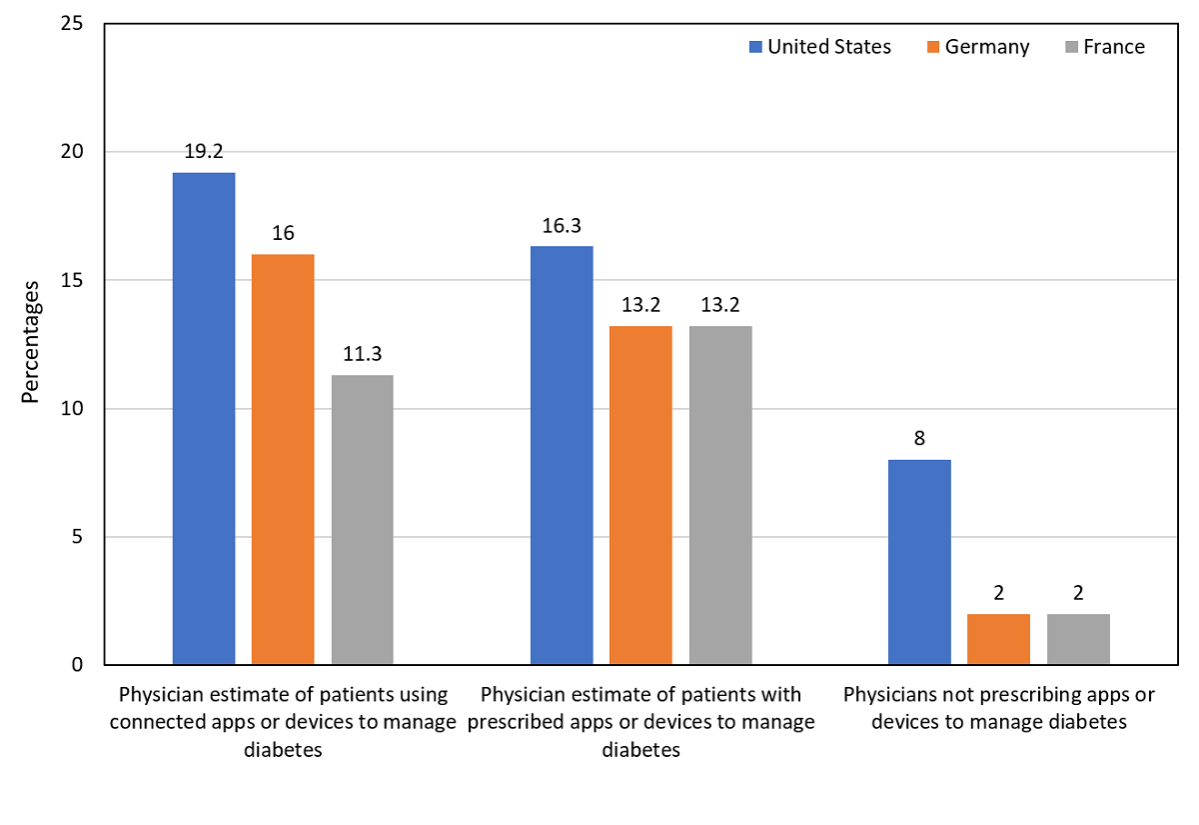
Physician estimates of patient use of connected devices.

Physicians thought that only a minority of their patients were using connected apps or devices to manage their diabetes (Figure 2). They also thought that a minority had been prescribed apps or devices to manage their disease, although few physicians reported *not* prescribing these technologies.

US physicians thought that a mean 50.3% (SD 20.5%; minimum-maximum 10-80) of their patients would benefit from a CES program. French physicians estimated a similar proportion would benefit (mean 51.2 [SD 23.6]; minimum-maximum 10-80), but the proportion estimated by German physicians was much lower (mean 24.5 [SD 12.5]; minimum-maximum 5-50).

#### Physician Attitudes Toward CES Programs

At least 80% of physicians in all 3 countries (n=28) agreed or strongly agreed that the use of a CES could help support their patients in managing their diabetes (Figure S1 in Multimedia Appendix 3). United States and German physicians reported less confidence in the ability of a CES program to strongly improve their own ability to support their patients.

#### Physician Interest in CES Programs

In the United States, at least half of the physicians were somewhat or very likely to recommend the use of a CES to their colleagues, to their patients, and within their practices (Figure S2 in Multimedia Appendix 3). This was also true for the other countries, except that half of German physicians were neutral or somewhat unlikely to recommend the use of a CES to their colleagues.

More than 80% of the physicians in all countries expressed interest in the following CES features for patient support: personalized data tracking (eg, blood glucose levels, meals, exercise, and sleep; n=31), medication reminders with recommended insulin doses (n=32), personalized recommendations (eg, for diet and exercise; n=29), and recording insulin doses (n=30; Figure S3 in Multimedia Appendix 3). The physicians expressed much less interest in the abilities of the patient to message them and other physicians, although American physicians expressed more interest in this feature than did clinicians in the other 2 countries.

The features of interest to physicians in supporting their management of patients with diabetes included the ability to review blood glucose trends, medication adherence data, behavioral data (eg, meals and exercise), and aggregated data about their patients (Figure S3 in Multimedia Appendix 3). Again, the abilities to message patients and other physicians were of much less interest, although a majority US physicians (53.3%; n=8) ranked the ability to message patients as important.

#### Physician-Reported Predictors of Patient Benefit From CES Use

**Figure 3:**
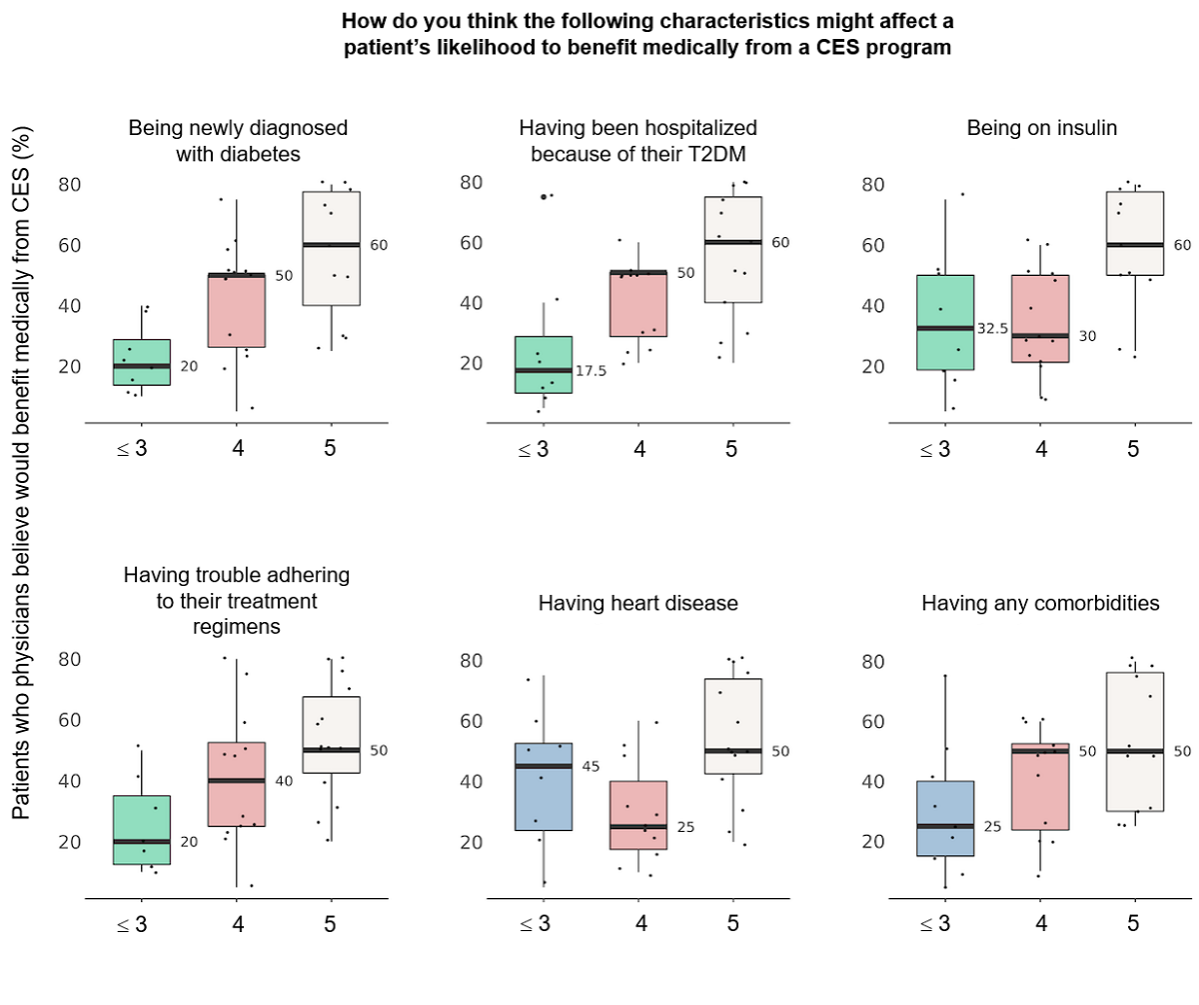
Physician-reported characteristics of patients likely to benefit medically from CES use. Assessed on a 5-point scale, where 0=physician belief that the patient would not likely benefit medically from CES use, 3=neutrality regarding likely medical benefit, and 5=physician belief that the patient would likely benefit medically from CES use. Horizontal lines indicate medians; boxes, the IQR; and vertical lines, the minimum and maximum. CES: connected ecosystem; T2DM: type 2 diabetes mellitus.

When presented with various clinical scenarios, physicians thought that sicker patients were more likely to benefit medically from CES use (Figure 3). Characteristics that were statistically significantly associated with physician belief in likely benefit included a new diagnosis of diabetes (*P*=.01), hospitalization because of diabetes (*P*=.01), current use of insulin (*P*=.02), medication nonadherence (*P*=.02), concomitant heart disease (*P*=.03), and any comorbid conditions (*P*=.048).

## Discussion

### Principal Results

**Figure 4:**
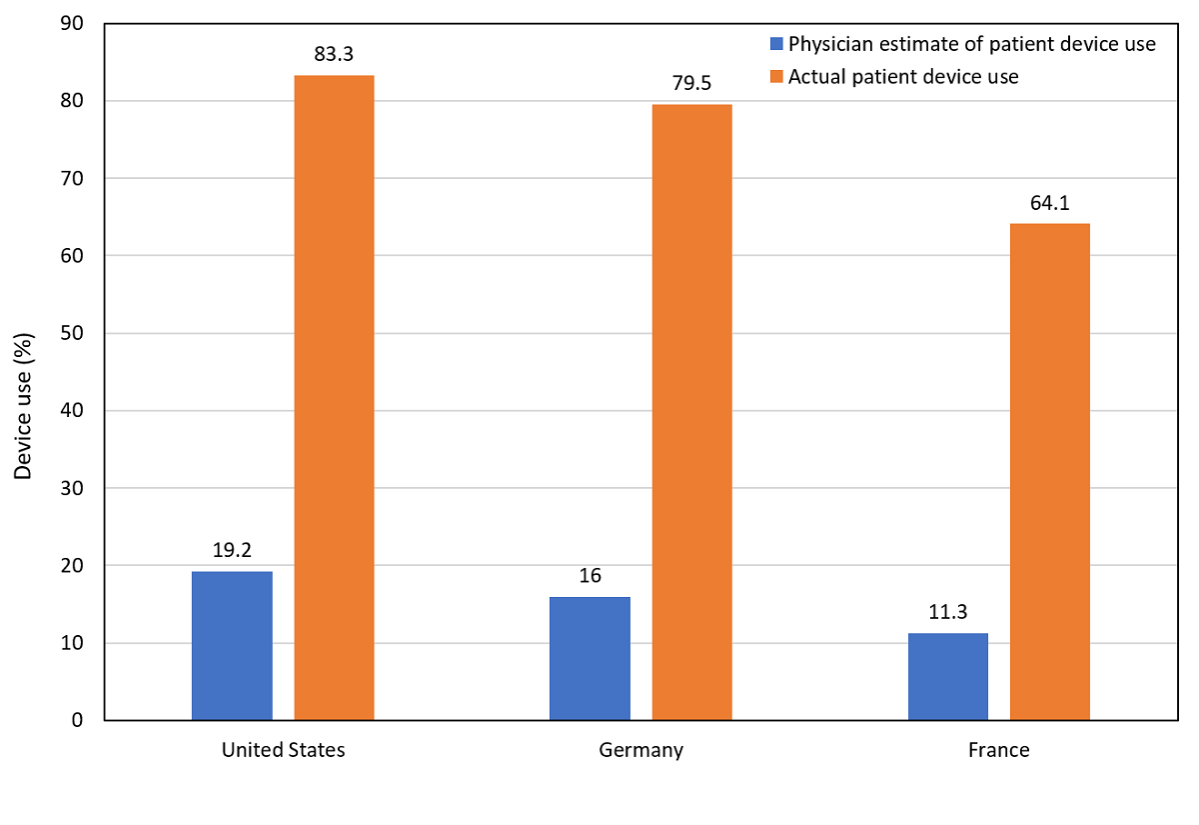
Physician perception of device use among patients versus actual patient device use.

In this cross-sectional study of 197 patients with insulin-dependent diabetes and 33 physicians caring for such patients, the majority, in all 3 countries, reported a wide variety of connected app and device use. Physician estimates of connected device and app use among patients were much lower (Figure 4), despite the fact that the vast majority reported prescribing connected apps to patients as a means of managing their diabetes. Physicians did report positive attitudes about the potential of CES use for managing diabetes and believed that patients who are sicker or less compliant with care might benefit most from a CES approach.

Although physicians in our study substantially underestimated the degree to which patients would be likely to use a CES, they did express interest in collaborative efforts with their patients and the potential of CES programs. Such collaborations have been shown to play a role in both adherence to and continuation of diabetes treatments [13,14], which in turn improves outcomes.

### CES Attributes

CESs, which incorporate disparate devices into a cohesive system, can give people with diabetes access to information about their condition and may improve the dialogue between patients and their health care practitioners. They have the advantages of being relatively inexpensive and easy to use and thus could represent a convenient addition to strategies for personalized, real-time management of diabetes and other conditions. The fact that both our patient and physician respondents indicated strong interest in CES use supports the development and deployment of such tools.

### Comparison With Prior Work

To our knowledge, this is the first study to examine interest in the use of a CES among patients, clinicians, and practice settings. Our findings from the 3 countries—combined with continued advances in and integration of devices such as smart insulin pens and caps and glucose monitoring systems, along with telemedicine and mobile health apps—lend support to the idea that a CES can contribute to improved, personalized, real-time self-care and clinical management of insulin-dependent diabetes globally [15].

### Limitations

This study is subject to the limitations inherent in all survey studies, including probable differences among the respondents in terms of question comprehension as well as recall bias and other biases. Further, persons who completed the survey may have been qualitatively different from nonrespondents. The data presented should be interpreted in the context of these limitations.

This study represents convenience-based cross-sections of persons with diabetes, and caregivers of such patients. Our findings therefore cannot reflect the entire communities of these cohorts. In particular, the very small sample sizes of the physicians in the 3 countries limit the generalizability of our results in this group. We also did not account for differences between countries in terms of recruitment techniques, demographic characteristics, physical and financial access to health care, and access to technologies in general, which could have skewed our analyses. For example, patients in the United States were far more likely than patients in the other countries to be employed full-time. These patients might be less likely to have time to devote to using a CES, as well as lacking insurance coverage of such systems. For physicians, there was a preponderance of endocrinologists in Germany and family medicine specialists in France. These specialties might differ in the degree of glycemic control desired, which might affect the frequency of CES usage.

All participants in the United States were existing members of the Achievement platform, reflecting a population already engaged with digital technology. Significant barriers persist in terms of access to digital health interventions and methods [16]. This study should serve as a basis for future evaluations that are tailored to reach patients and health care practitioners from more diverse backgrounds more effectively, including those not currently using digital technologies, to better assess the acceptance and usage of CES.

### Conclusions

In these 3 countries, most patients reported either past or current use of connected devices to manage their T2DM. They also expressed strong interest in the use of a CES for this purpose. The physicians surveyed agreed that their patients would benefit from CES use, particularly patients who were sicker or noncompliant with treatment. However, their estimates of patient use of devices were much lower than actual rates of use. If future hands-on studies of CES prove their effectiveness, both patients with diabetes and their physicians might achieve improvements in self-management and clinical care for the disease.

## Appendix

Multimedia Appendix 1 Survey instruments.

Multimedia Appendix 2 Patient survey results.

Multimedia Appendix 3 Physician survey results.

## Abbreviations

CES: connected ecosystem
T2DM: type 2 diabetes mellitus

## Data Availability

Qualified researchers may request access to the aggregate results and related study documents including the study report, study protocol with any amendments, blank case report form, statistical analysis plan, and data set specifications. Further details on Sanofi’s data sharing criteria, eligible studies, and process for requesting access can be found at the Vivli—Center for Global Clinical Research Data website [17].
