# Supplementary material for: Patient and Physician Perspectives on the Use of a Connected Ecosystem for Diabetes Management: International Cross-Sectional Observational Study
Source: JMIR Form Res. 2023 Nov 30;7:e47145. doi: 10.2196/47145 (PMC10722356; doi:10.2196/47145)
Supplement: Multimedia Appendix 1 [file formative_v7i1e47145_app1.pdf]

CES
Patient Case Report Form (CRF)

Thank you for participating in this important study about your experience with diabetes and ways to manage your diabetes. This survey has 4 sections and should take about 20-25 minutes to complete.

Demographics
The first section asks some basic questions about you and your health.

1. Which racial or ethnic group(s) do you identify with? Please select all that apply.
    a. American Indian or Alaskan Native
    b. Asian
    c. Black, African American, African descent, or from the African diaspora
    d. Hispanic, Latino, or Spanish
    e. Middle Eastern or North African
    f. Native Hawaiian or Other Pacific Islander
    g. White
    h. Another race or ethnicity
    i. I prefer not to answer [mutually exclusive]

2. What is your height?
    feet _______ [*range: 3-7]*
    inches _______ [*range: 0-11]*

3. What is your current weight?
    lbs _______ [*range: 50-1000]*

4. Do you currently have any form of health insurance? [has_insurance]
    a. Yes
    b. No
    c. I don’t know / I’m not sure
    d. I prefer not to answer

[IF has_insurance == “Yes”, ask:]

5. What best describes your current health insurance status? Please select all that apply.
    a. I’m insured through a current or former employer or union (of myself or a family member)
    b. I purchase insurance directly from an insurance company (by myself or a family member)

c. I'm insured through Medicare, for people 65 and older, or people with certain disabilities
d. I'm insured through Medicaid, Medical Assistance, or any kind of government-assistance plan for those with low incomes or disability
e. I'm insured through TRICARE or other military health care
f. I'm insured through Indian Health Service
g. I don't know / I'm not sure
h. I prefer not to answer [mutually exclusive]
i. Something else

6. What is the highest level of education that you have completed?
   a. No formal education
   b. Some elementary and/or middle school (grades 1-8)
   c. Some high school (no diploma or GED)
   d. High school degree or equivalent (for example, GED)
   e. Some college, no degree
   f. Trade/technical/vocational training
   g. Associate's degree (for example, AA, AS)
   h. Bachelor's degree (for example, BA, BS)
   i. Master's degree (for example, MA, MS, MEd)
   j. Professional degree (for example, MD, DDS, DVM)
   k. Doctorate (for example, PhD, EdD)
   l. I prefer not to answer

7. What is your current employment status? Please select all that apply.
   a. Employed full-time (35 or more hours per week)
   b. Employed part-time (34 or less hours per week)
   c. Working as a homemaker or caregiver (unpaid)
   d. Not currently employed
   e. Currently looking for employment
   f. Unable to work
   g. Receiving disability/governmental support
   h. Student
   i. Retired
   j. I prefer not to answer [mutually exclusive]

8. Which of the following best describes your current relationship status? Please select all that apply.
   a. Divorced
   b. In a committed partnership / living with partner(s)
   c. Married / in a domestic partnership or civil union
   d. Separated

e. Single
f. Widowed
g. I prefer not to answer [mutually exclusive]

9. Do you currently have any dependents (for example, children under the age of 18 or other family members that rely on you for support)?
    a. Yes
    b. No

10. Do you have any adult children (that is, children over the age of 18)?
    a. Yes
    b. No

11. What is the zip code where you live most of the time? *[single textbox interface, validated]*
    a. [5-digit number entry in the US]

12. Do you currently use any kind of walking assistance device (for example, a wheelchair, walker, or cane)?
    a. Yes, I use a walking assistance device nearly all of the time
    b. Yes, I only use a walking assistance device occasionally
    c. No, I never use any walking assistance devices

Medical History, Diabetes Management, and Healthcare Utilization
The second section asks questions about your medical history, including information about your Type 2 Diabetes.

13. Have you been diagnosed with any of the following health conditions? Please select all that apply.
    a. Arthritis (osteoarthritis or rheumatoid)
    b. Asthma
    c. Cancer of any kind
    d. Heart / cardiovascular disease (CVD)
    e. Chronic pain
    a. Chronic obstructive pulmonary disease (COPD)
    a. Glaucoma
    b. Heart failure
    b. High cholesterol
    c. High blood pressure (hypertension)
    d. Kidney disease
    e. Liver disease (for example, cirrhosis, hepatitis B, hepatitis C)
    f. Mental health conditions of any kind (for example, major depressive disorder, anxiety)

g. Neurological conditions (for example, epilepsy)
h. Obesity
i. Peripheral arterial disease
j. Stroke
k. None of the above [mutually exclusive]

14. Do you currently smoke cigarettes?
    a. Yes, I smoke regularly (daily or almost daily)
    b. Yes, I smoke occasionally
    c. No, I used to smoke regularly but I don’t currently (smoked over 100 cigarettes total in my lifetime)
    d. No, I have never smoked

15. How often do you drink alcohol, on average?
    a. Never
    b. Rarely (a few times a year)
    c. Occasionally (a few times a month)
    d. Frequently (a few times a week)
    e. Daily

16. Approximately how old were you when you were diagnosed with Type 2 Diabetes?
    a. [Number entry]

17. In addition to your insulin, do you use any of the following to manage your Type 2 Diabetes? Please select all that apply.
    a. With oral medications
    b. With non-insulin injectable medications
    c. With diet and exercise
    d. Something else
    e. None of the above [mutually exclusive]

18. Which of the following types of insulin have you *ever* used to manage your Type 2 Diabetes? Please select all that apply. [insulin_ever_used]
    a. Short-acting (for example, regular, lispro, aspart, glulisine)
    b. Intermediate-acting (basal, for example, NPH)
    c. Long-acting (basal) analog insulin (for example, basaglar, glargine, detemir, degludec)
    d. Multi-drug injection treatment (for example, a combination of basal insulin and short-acting insulin)
    e. Something else

19. Which of the following types of insulin do you *currently* use to manage your Type 2 Diabetes? Please select all that apply.
    a. [Pull forward from insulin_ever_used]

20. How often do you *currently* use insulin to manage your Type 2 Diabetes?
    a. After every meal
    b. Multiple times per day
    c. About once per day
    d. Less than once per day

21. Have you *ever* used any of the following connected health devices to help manage your Type 2 Diabetes? Please select all that apply. [connected_devices_ever_used]
    a. Smart blood pressure monitors
    b. Smart watches
    c. Artificial pancreas
    d. Smart socks
    e. Dedicated apps / websites (for example, Glooko)
    f. Flash / continuous blood sugar (glucose) monitors
    g. Smart ECGs (for example, via an Apple Watch)
    h. Smart scale (for example, Withings)
    i. Something else
    j. I've never used any connected health devices to help manage my Type 2 Diabetes [mutually exclusive]

[if connected_devices_ever_used is not "I've never used any connected health devices"]

22. On a scale of 1-10, where 1 is not helpful at all and 10 is the most helpful, how would you rate the helpfulness of your previously used connected health devices to manage your Type 2 diabetes?
    a. [pull forward from connected_devices_ever_used, matrix question w/ 1-10]

[if connected_devices_ever_used is not "I've never used any connected health devices"]

23. Do you *currently* use any of the following connected health devices to help manage your Type 2 Diabetes? Please select all that apply.
    a. [pull forward from connected_devices_ever_used]
    b. I don't currently use any connected health devices to help manage my Type 2 Diabetes [mutually exclusive]

24. How often do you currently measure your blood sugar levels?
    a. Multiple times per day
    b. About once per day
    c. Multiple times per week
    d. About once per week

   e. Less than once per week

25. Is your Type 2 diabetes well-controlled (for example, has your day-to-day blood sugar measurement been steady over the past month)?
    a. Yes
    b. No
    c. I don't know / I'm not sure

26. What was your last HbA1c level?
    The HbA1c test evaluates the average amount of glucose in the blood over the last 2 to 3 months by measuring the percentage of glycated hemoglobin in the blood.
    a. 6.0 or less
    b. Between 6.1 and 6.5
    c. Between 6.6 and 7.5
    d. Greater than 7.5
    e. I don't know / I'm not sure

27. Do you have any of the following personal goals to help you manage your Type 2 Diabetes? Please select all that apply. [personal_goals]
    a. Increasing physical activity / exercise
    b. Eating more healthily
    c. Reducing stress
    d. Reducing alcohol consumption
    e. Quitting smoking
    f. Being better about taking my medications on time
    g. Better understanding the relationship between my blood sugar levels and my behaviors (for example, the things I eat, my activities)
    h. Another health-related goal
    i. I do not have any personal goals related to my Type 2 Diabetes management [mutually exclusive]

[IF personal_goals is NOT "I do not have any personal goals"]

28. Which of your Type 2 Diabetes management goals do you wish you had more help with? Please select all that apply.
    a. [Pull forward from personal_goals]
    b. I don't need help achieving any of my Type 2 Diabetes management goals [mutually exclusive]

29. Do you have a primary care physician?
    a. Yes
    b. No
    c. I don't know / I'm not sure

30. About how many times per year do you visit a doctor's office for yourself specifically regarding your Type 2 Diabetes?
    a. Dropdown with numbers [0-11, 12+]

31. About how many times per year do you visit an urgent care clinic for yourself regarding your Type 2 Diabetes?
    a. Dropdown with numbers [0-11, 12+]

32. About how many times in the past year have you been to the emergency room (ER) for a medical problem of your own? Don't include ER visits where you were bringing someone else and didn't have your own reason to go there. [num_ER_visits]
    a. Dropdown with numbers [0-11, 12+]

[IF num_ER_visits≠0, ASK:]

33. How many of these ER visits were because your blood sugars were too low?
    a. Dropdown with numbers [0-11, 12+]

[IF num_ER_visits≠0, ASK:]

34. How many of these ER visits were because your blood sugars were too high?

Dropdown with numbers [0-11, 12+]

35. Have you ever been hospitalized? [hospitalization]
    a. Yes
    b. No

[IF hospitalization=YES, ASK:]

36. How many times in the past year have you been hospitalized? [hospitalization_last_year]
    a. Dropdown with numbers [0-11, 12+]

[IF hospitalization_last_year>0, ASK:]

37. How many of these hospitalizations overall were because your blood sugar levels were too low?
    a. Dropdown with numbers [0-11, 12+]

[IF hospitalization_last_year>0, ASK:]

38. How many of these hospitalizations overall were because your blood sugar levels were too high?
    a. Dropdown with numbers [0-11, 12+]

39. Overall, how satisfied are you with your current Type 2 Diabetes management?
    a. Very satisfied

b. Somewhat satisfied
c. Neither satisfied nor dissatisfied
d. Somewhat dissatisfied
e. Very dissatisfied

Lifestyle
The next section asks you questions about your daily life.

40. On a scale from 1 to 5, how would you rate each of the following aspects of your life?

	1 Poor	2 Fair	3 Good	4 Very good	5 Excellent
Having a balanced/healthy diet					
Your level of exercise/physical activity					
Your sleep quality					
Your overall health and wellbeing					
Your quality of life					

41. On average, how many days per week do you exercise for at least 30 minutes?
    a. Dropdown with 0-7

42. On average, how many hours of sleep do you get on a <u>weeknight</u> (Sunday-Thursday)?
    a. [number entry between 0 and 24, allow decimals]

43. On average, how many hours of sleep do you get on a <u>weekend</u> night (Friday-Saturday)?
    a. [number entry between 0 and 24, allow decimals]

Potential Diabetes Management Program
The last section asks you questions about a potential digital program to help you manage your Type 2 Diabetes.

A Connected EcoSystem (CES) is a tool that you could use to improve your Diabetes management. It would be designed to help you keep your blood sugar under control. It could include using a digital device to track your insulin delivery and helping you monitor your blood sugar (glucose) levels. You could also talk with your healthcare provider about your treatment with the CES tool.

44. How likely would you be to participate in the program described above?

a. [scale from 0: not at all likely to 10: very likely]

45. How important would the following be for you to know if you were deciding whether to participate in the program described above? [important_factors]
    a. How much it would cost me
    b. How much time it would take
    c. How personalized the experience would be
    d. Whether my doctor recommended it
    e. What my friends or family thought of it
    f. Whether it shared information with my doctor
    g. Something else

    [Matrix question: scale from 0: not at all important to 10: very important]

[IF personal_goals is NOT “I do not have any personal goals”]

46. Which of your Type 2 Diabetes management goals do you think the program described above could help you achieve? Please select all that apply.
    a. [Pull forward from personal_goals]
    b. I don’t think the program described above could help me achieve any of my personal Type 2 Diabetes management goals [mutually exclusive]

47. Which of the following features would you most want to see in a digital health program designed to support your Type 2 Diabetes management? Please select all that apply.
    a. The ability to talk to the doctors I see most often about my Type 2 Diabetes
    b. The ability to talk to a mental health professional
    c. The ability to talk to other people with Type 2 Diabetes
    d. General information about Type 2 Diabetes management
    e. The ability to track my medication usage
    f. The ability to track my blood sugar trends
    g. The ability to track my meals
    h. The ability to track other health-related information (for example, sleep, stress)
    i. Personalized recommendations (for example, food or exercise recommendations)
    j. Progress reports on my personal goals (e.g., changes in weight, exercise, nutrition, or smoking)
    k. Encouragement for managing my Type 2 Diabetes
    l. Medication reminders
    m. Another potential feature
    n. I would never be interested in a digital health program designed to support my Type 2 Diabetes management [mutually exclusive]

CES Survey
Physician Case Report Form (CRF)

Thank you for participating in this important study about your Type 2 diabetes patients and ways to help your patients manage their diabetes. This survey should take about 20-25 minutes to complete.

1. What sex were you assigned at birth (for example, on your birth certificate)?
    a. Female
    b. Intersex
    c. Male
    d. I prefer not to answer
2. What is your current gender? Please select all that apply.
    a. Female
    b. Gender Queer
    c. Male
    d. Transgender Female/Trans Woman/MtF
    e. Transgender Male/Trans Man/FtM
    f. A gender not listed here
    g. I prefer not to answer
3. What is your medical degree?
    a. MD
    b. DO
    c. Other
4. What is your medical specialty?
    a. Family medicine
    b. Internal medicine
    c. Pediatrics
    d. Endocrinology
    e. Other
5. How many years have you been practicing medicine?
    a. 0-10
    b. 11-20
    c. 21-30
    d. 31+

6. Approximately how many patients with Type 2 Diabetes Mellitus (T2DM) have you treated in the past 12 months?
    a. [number entry]

7. Approximately what percentage of your patients with T2DM require combination therapy?
    a. [number entry between 0 and 100]

8. Approximately what percentage of your patients with T2DM are treated with non-insulin injectables?
    a. [number entry between 0 and 100]

9. Approximately what percentage of your patients with T2DM have health insurance?
    a. [number entry between 0 and 100]

10. Approximately what percentage of your patients with T2DM have at least one comorbidity?
    a. [number entry between 0 and 100]

11. Approximately what percentage of your patients with T2DM have kidney disease?
    a. [number entry between 0 and 100]

12. Approximately what percentage of your patients with T2DM require dialysis?
    a. [number entry between 0 and 100]

13. Approximately what percentage of your patients with T2DM have ischemic heart disease?
    a. [number entry between 0 and 100]

14. Approximately what percentage of your patients with T2DM have peripheral arterial disease?
    a. [number entry between 0 and 100]

15. Approximately what percentage of your patients with T2DM have been hospitalized because of either hypoglycemia or hyperglycemia?
    a. [number entry between 0 and 100]

16. Approximately what percentage of your patients with T2DM have had a stroke?
    a. [number entry between 0 and 100]

17. Approximately what percentage of your patients with T2DM have achieved glycemic control?

   a. [number entry between 0 and 100]

18. Approximately what percentage of your patients with T2DM have connected apps or devices to help them manage their Diabetes (e.g., Glooko)?
    a. [number entry between 0 and 100]

19. For approximately what percentage of your patients with T2DM have you *prescribed* connected apps or devices to help them manage their Diabetes (e.g., Glooko)?
    a. [number entry between 0 and 100]

20. Approximately what percentage of your patients with T2DM have trouble adhering to their medications?
    a. [number entry between 0 and 100]

21. Approximately what percentage of your patients with T2DM have trouble adhering to your recommended behavioral changes (e.g., diet, exercise)?
    a. [number entry between 0 and 100]

22. Approximately what percentage of your patients with T2DM are overweight or obese?
    a. [number entry between 0 and 100]

23. On average, approximately how often *do* you see your patients with T2DM to discuss their Diabetes?
    a. Multiple times per month
    b. About once per month
    c. Multiple times per year
    d. About once per year
    e. Less than once per year

24. On average, approximately how often would you *like* to see your patients with T2DM to discuss their Diabetes management?
    a. Multiple times per month
    b. About once per month
    c. Multiple times per year
    d. About once per year
    e. Less than once per year

25. On average, approximately how often do your patients with T2DM go to the emergency room for their T2DM?
    a. Multiple times per month
    b. About once per month
    c. Multiple times per year

d. About once per year
e. Less than once per year

26. On average, approximately how often do you adjust prescriptions for your patients with T2DM?
    a. Multiple times per month
    b. About once per month
    c. Multiple times per year
    d. About once per year
    e. Less than once per year

A Connected Ecosystem (CES) is a network of tools and capabilities to support patients requiring improved glycaemic control. For a patient, this may mean using a digital device to track insulin delivery or to monitor his/her blood glucose levels, or provide a digital support platform to discuss with his/her HCP on appropriate therapy. For physicians, a CES can help improve decision making, practice efficiency, and provide effective care for patients. The goal of the CES is to help identify patients at higher risk for worse disease outcomes and improve disease management.

Given the information above, how strongly do you agree or disagree with the statements below (1= strongly disagree, 5 = strongly agree)?

	Strongly disagree 1	Disagree 2	Neutral 3	Agree 4	Strongly agree 5
27. I feel that CES could strongly improve *my* ability to support my patients with T2DM in managing their Diabetes					
28. I feel that CES could strongly support my patients with T2DM in managing their *own* Diabetes					

1. Given the information above, how likely are you to do each of the following (1= very unlikely, 5 = very likely)?

	Very unlikely 1	Somewhat unlikely 2	Neutral 3	Somewhat likely 4	Very likely 5
Recommend CES to your patients					

Advocate for using CES within your practice					
Recommend colleagues to use CES as part of their treatment for patients with T2DM					

29. Given the information included above, approximately what percentage of your patients with T2DM do you think would medically benefit from the CES program?
    a. [number entry between 0 and 100]

30. How do you think following characteristics might affect a patient's likelihood to medically benefit from the CES program described above?[characteristics_affect_likelihood]
    a. Being newly diagnosed with Diabetes
    b. Having insurance
    c. Having *any* comorbidities
    d. Having *more than one* comorbidity
    e. Having kidney disease
    f. Having heart disease
    g. Having a disability
    h. Having been hospitalized because of their T2DM
    i. Not having achieved glycemic control
    j. Having trouble adhering to their treatment regimens
    k. Being *younger* than the average patient with T2DM
    l. Being *older* than the average patient with T2DM
    m. Being familiar with technology
    n. Being overweight or obese
    o. Being on insulin
    p. Needing combination therapy
    q. Struggling to maintain healthy diet and exercise habits
    r. Struggling to remember to take their medications
    s. Struggling with motivation to manage their Diabetes
    t. Being married
    u. Having dependents
    v. Having adult children
    w. Living close to a hospital
    x. Another patient social or demographic characteristic
    y. Another patient clinical characteristic

[Matrix question: scale from 0: less likely to benefit to 3: neutral/no effect to 5: more likely to benefit]

31. Given the information included above, approximately what percentage of your patients with T2DM do you think would *use* the CES program?
    a. [number entry between 0 and 100]

32. Which of the following features would you want such a program to include to best support your patients with T2DM?
    a. The ability to message you
    b. The ability to message other physicians
    c. General information about Diabetes management
    d. Personal data tracking (e.g., blood sugar trends, meals, exercise, sleep, medications)
    e. Personalized recommendations (e.g., diet, exercise)
    f. Medication reminders with recommended dose of insulin injection
    g. Recording of dose of insulin injected
    h. Another potential feature

33. Which of the following features would you want such a program to include to best support *your* workflows?
    a. The ability to message your patient
    b. The ability to message other physicians your patient has seen
    c. The ability to review your patient's medication adherence
    d. The ability to review your patient's blood sugar trends
    e. The ability to review your patient's behavioral data (e.g., activity data, meals, exercise)
    f. The ability to review aggregated information across your patients with T2DM
    g. Another potential feature
