# Supplementary material for: Patient and Physician Perspectives on the Use of a Connected Ecosystem for Diabetes Management: International Cross-Sectional Observational Study
Source: JMIR Form Res. 2023 Nov 30;7:e47145. doi: 10.2196/47145 (PMC10722356; doi:10.2196/47145)
Supplement: Multimedia Appendix 2 [file formative_v7i1e47145_app2.pdf]

Figure S1. US Participants reported common use of connected devices and high utility for their use

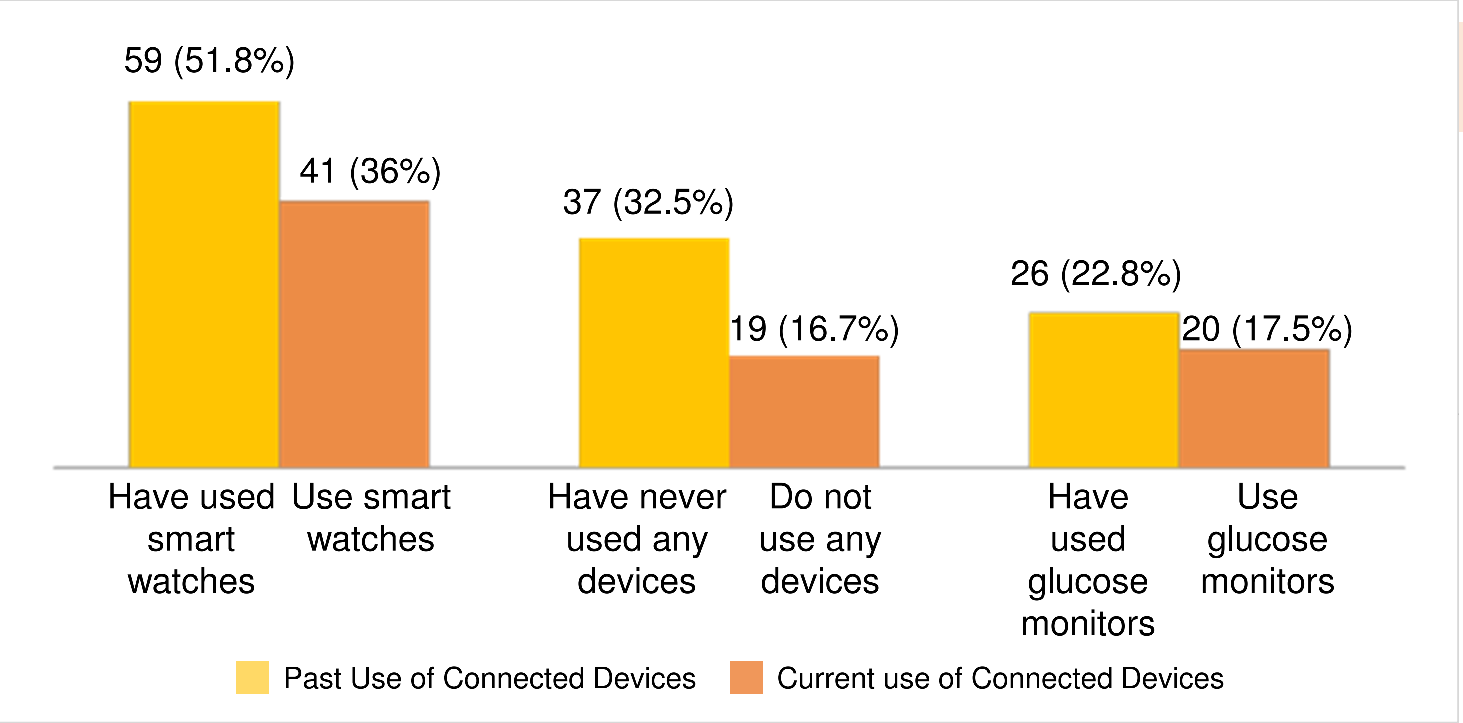

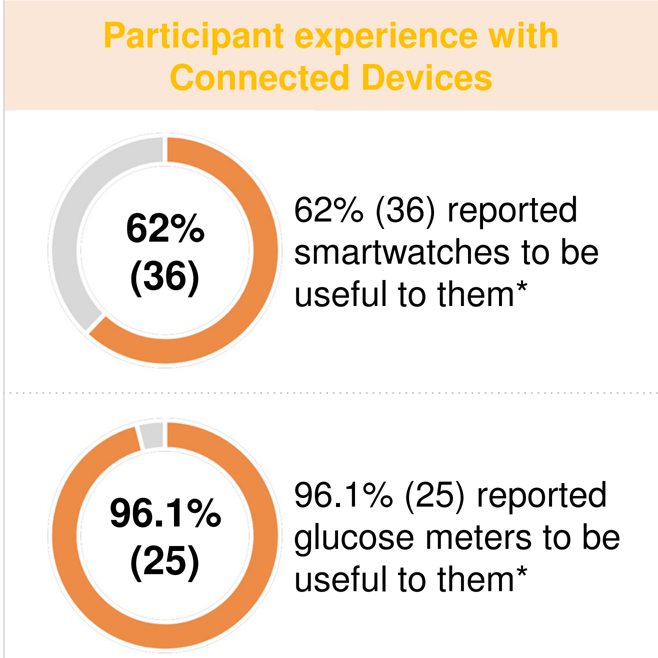

* Usefulness defined as a score of at least 7 on a 10-point scale. Percentages computed out of total participants that reported using each type of device.

Figure S2. Compared with the U.S., German participants reported more use of glucose monitors and connected apps, but less utility from these devices

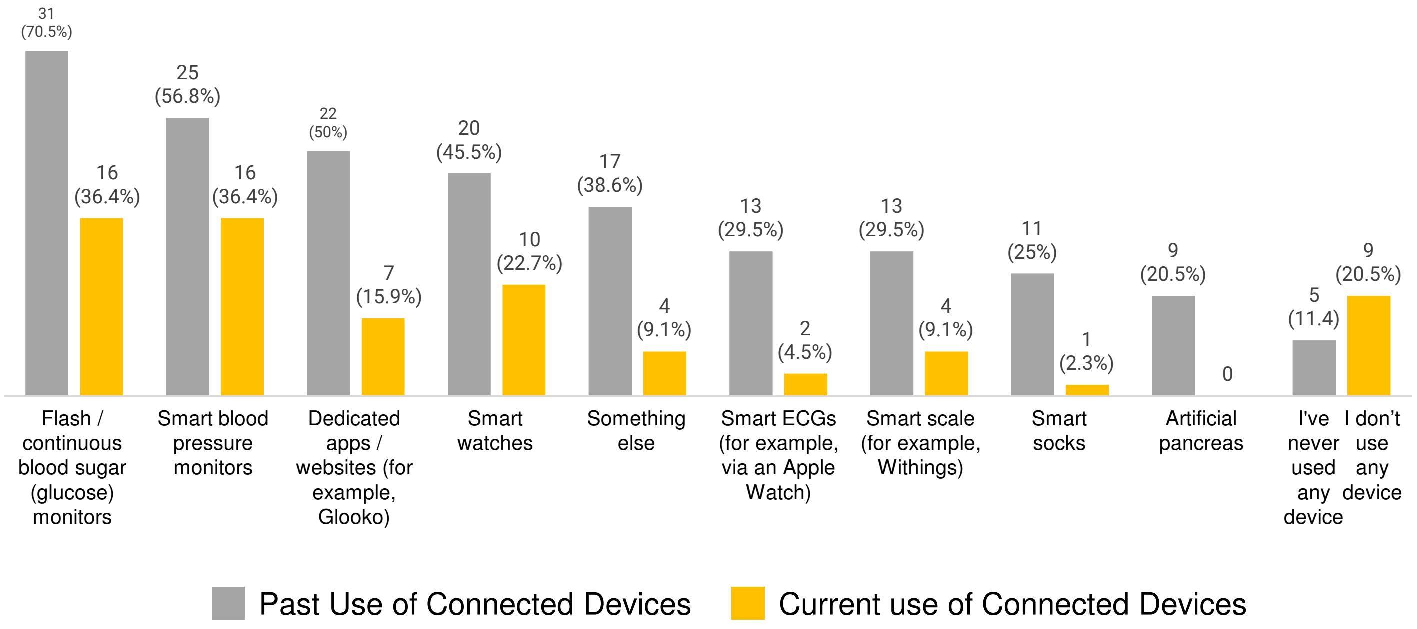

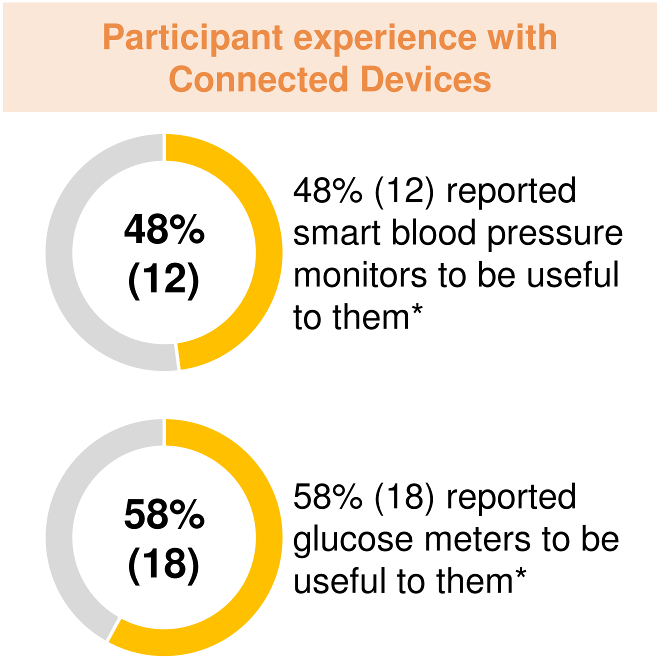

* Usefulness defined as a score of at least 7 on a 10-point scale. Percentages computed out of total participants that reported using each type of device.

Figure S3. French participants reported frequent use of glucose meters and connected apps, but less utility from them

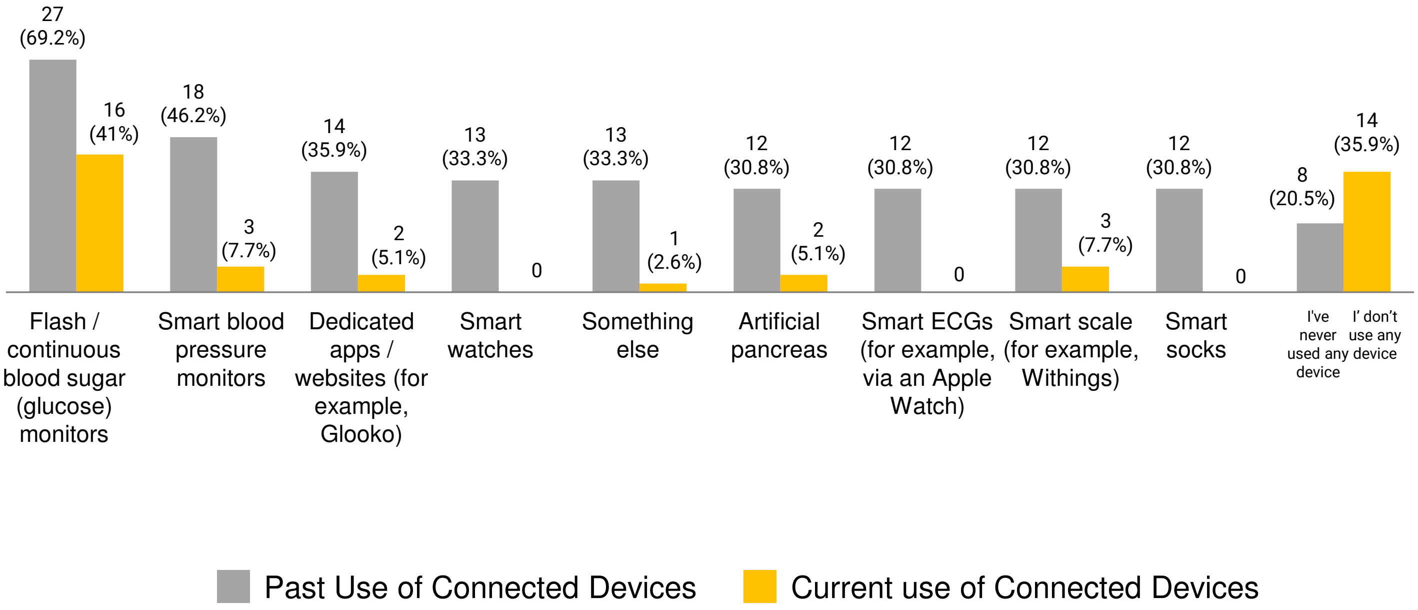

Participant experience with Connected Devices

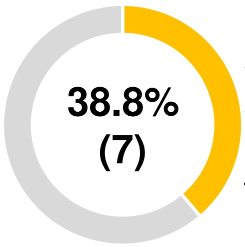

38.8% (7) reported smart blood pressure monitors to be useful to them*

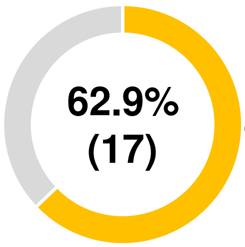

62.9% (17) reported glucose meters to be useful to them*

* Usefulness defined as a score of at least 7 on a 10-point scale. Percentages computed out of total participants that reported using each type of device.

Figure S4. US participants expressed interest in healthy behaviors and better tracking of blood glucose

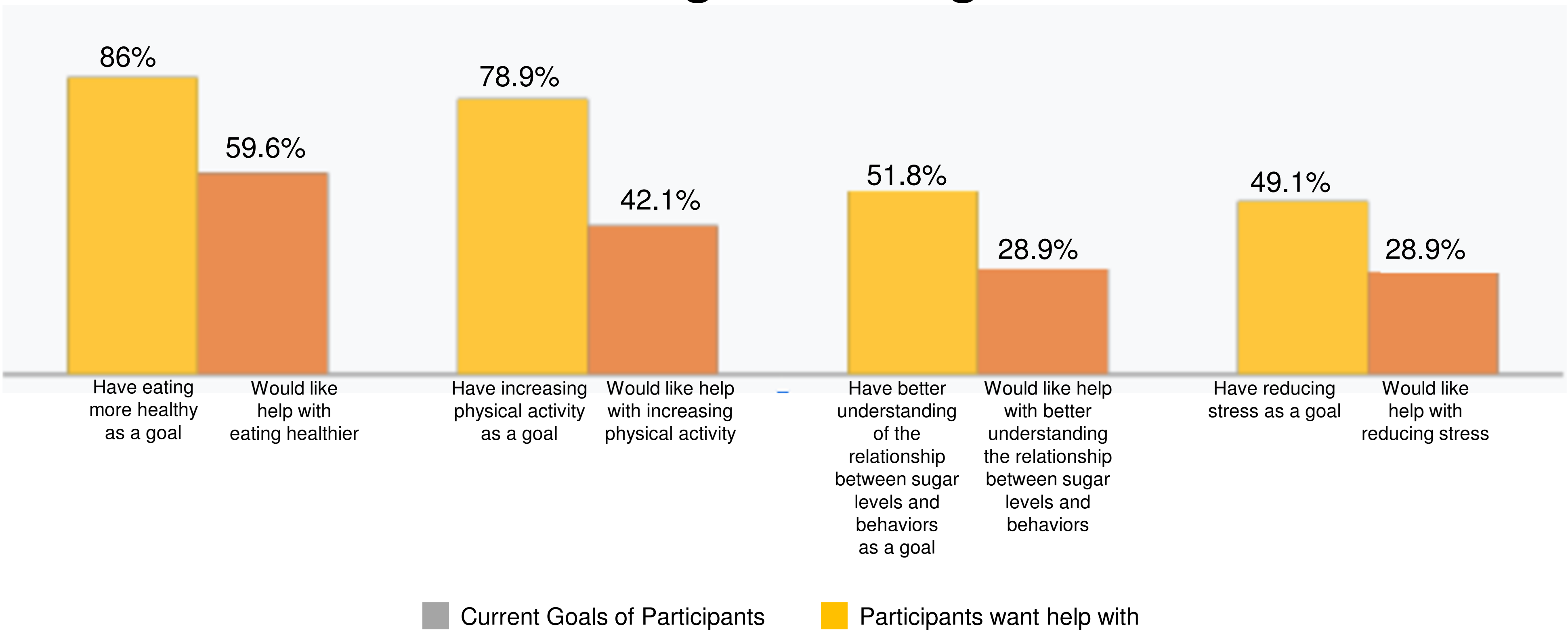

Figure S5. Like the U.S., German participants wanted help with healthy behaviors, medication compliance, and reducing stress

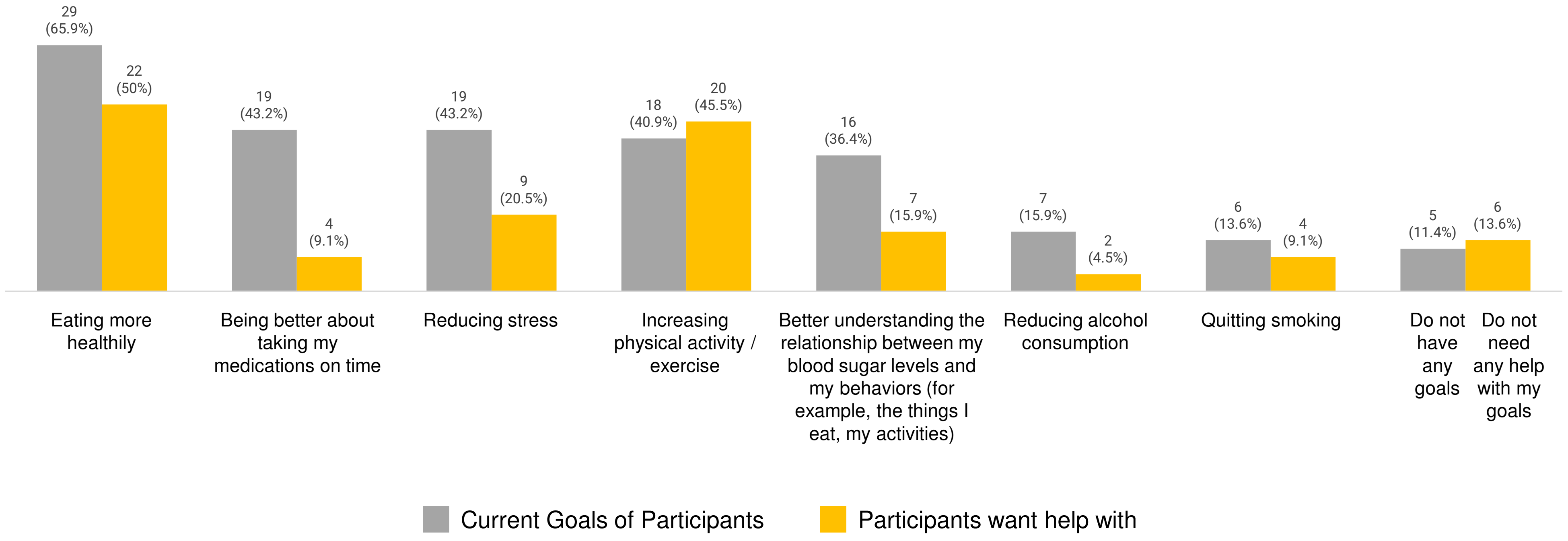

Figure S6. French participants reported similar health goals to US participants

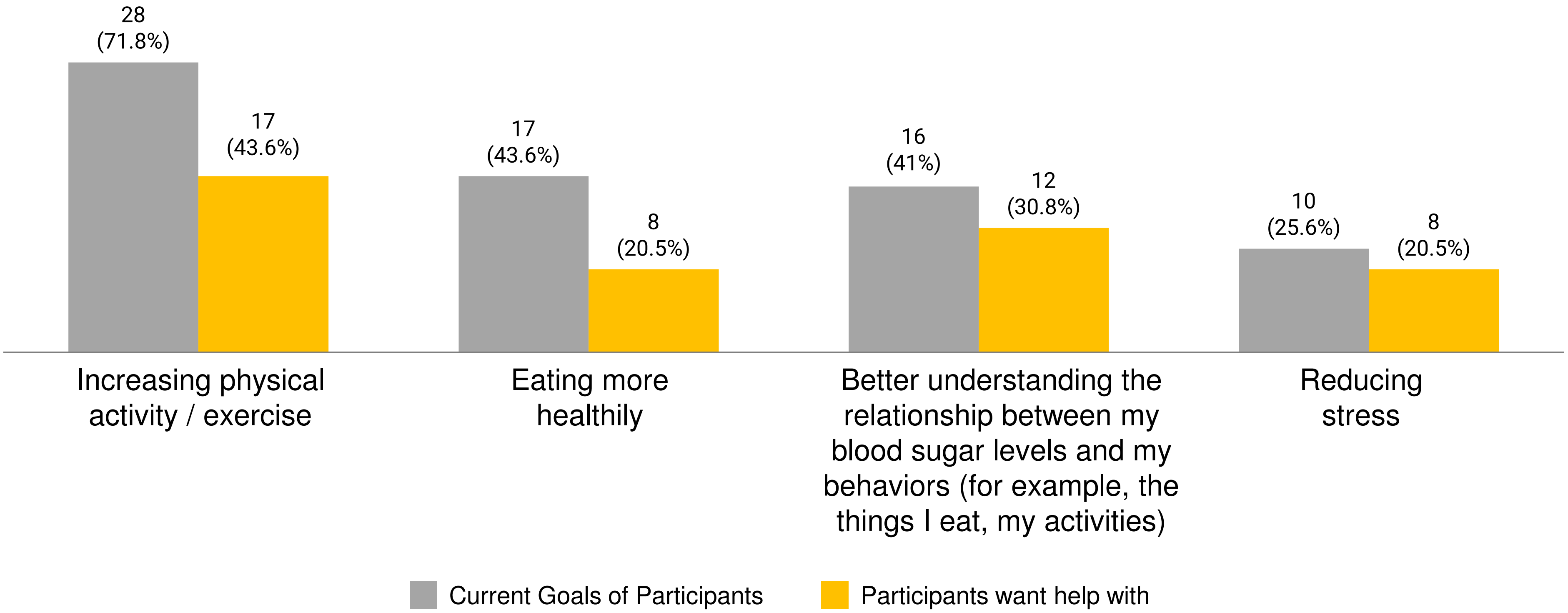

Figure S7. US participants wanted a CES program that features personalized tracking of behaviors, medications, and T2DM outcomes

Program Goals Expectations | Patients would like help with

Participants reported the CES program could help them achieve

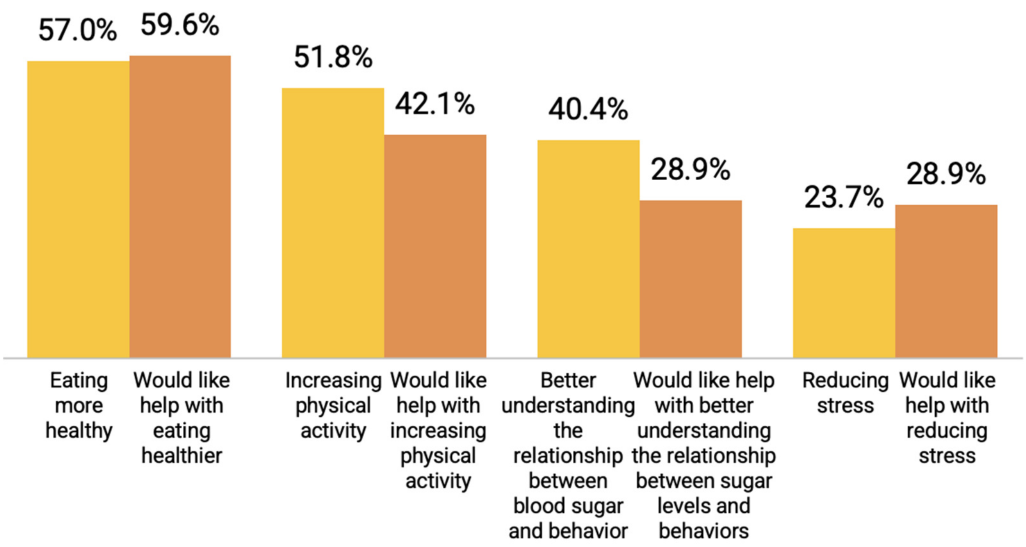

Program Needs

Participants reported they wanted the following features the most in a digital program to manage T2D (see table)

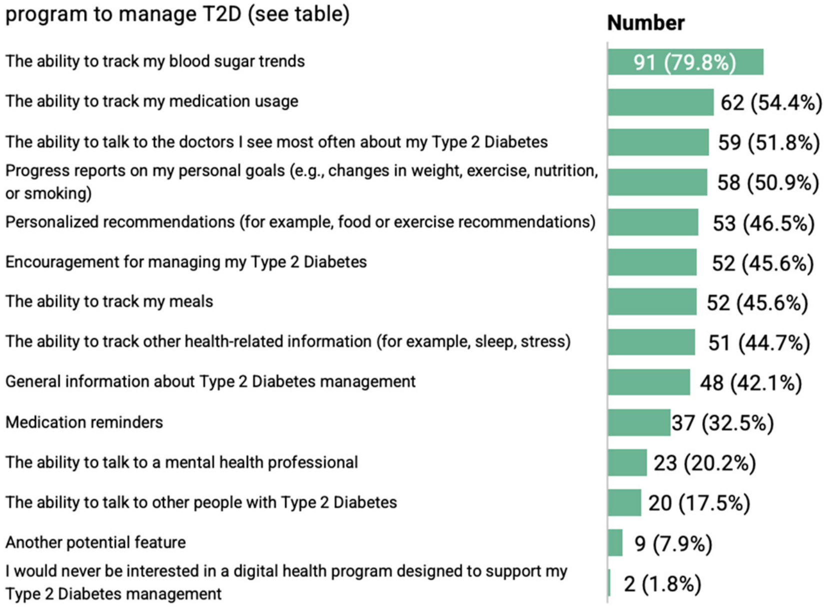

Figure S8. Similar to the US, German participants wanted a CES program that offers tracking of behaviors, medications and blood glucose

Program Goals Expectations | **Patients would like help with**

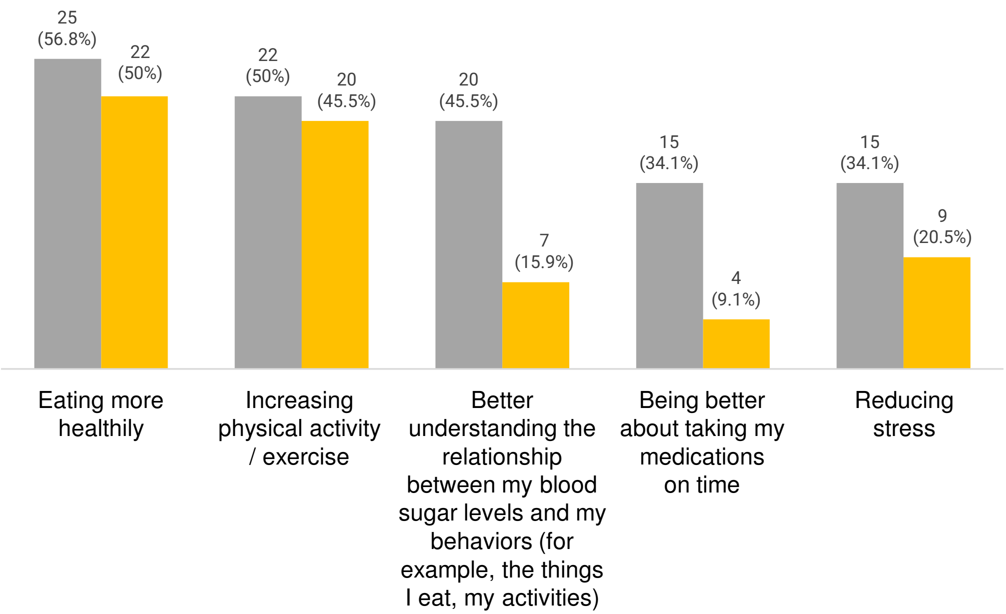

Program Needs

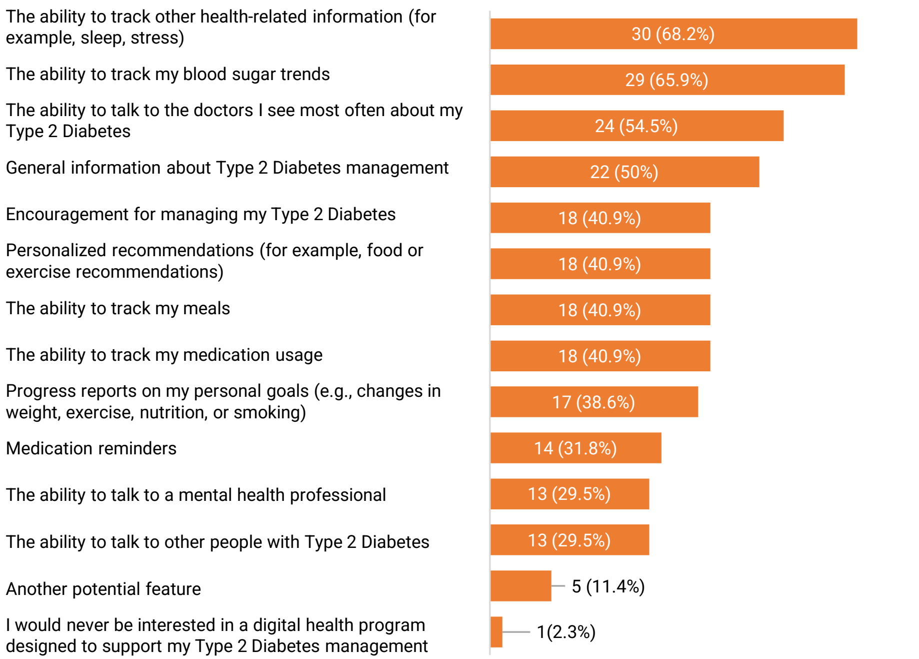

Figure S9. French participants wanted a CES that provides information and encourages communication with physicians in addition to tracking

Program Goals Expectations | **Patients would like help with** | **Program Needs**

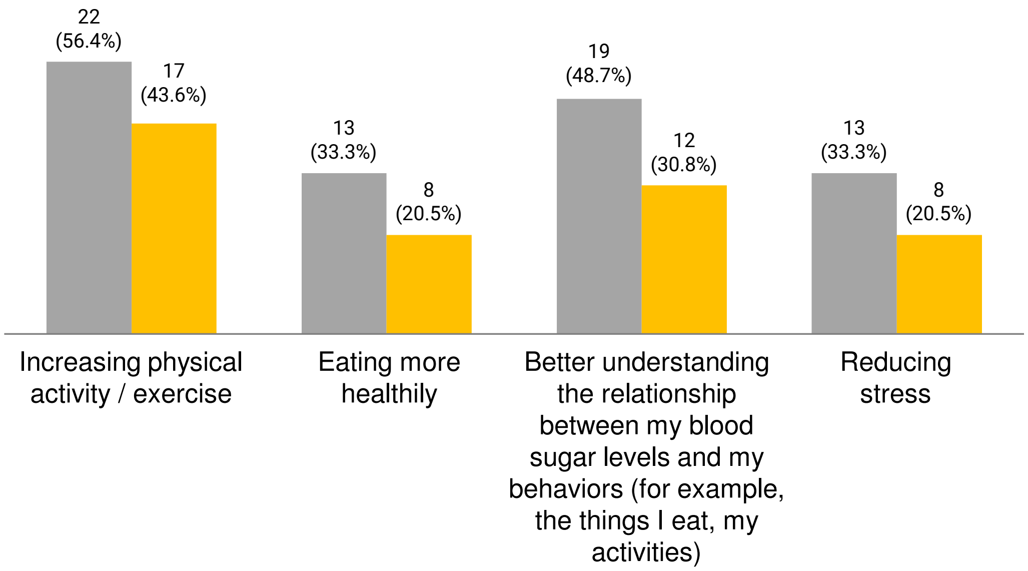

Participants reported they wanted the following features the most in a digital program to manage T2DM (see table)

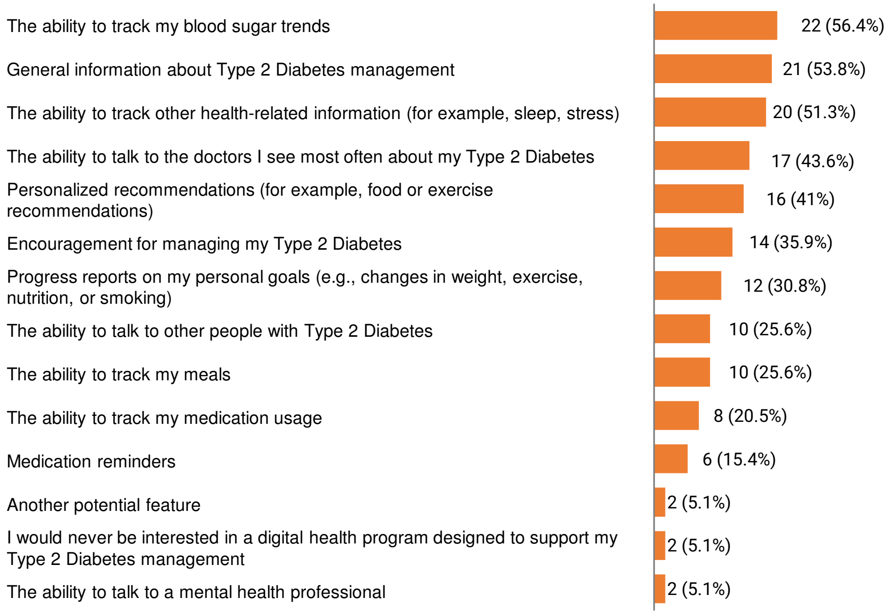

Figure S10. Factors affecting whether to participate in a CES program: U.S.

Cost,* Medication reminders,† and tracking of health related info† were the largest factors influencing **willingness to participate in a US CES program**

US participants who responded they would never be interested in a digital health program for T2DM* were more than 4 times less likely to participate in a CES program

* at 0.05 stat. sig. level without multiplicity correction
† at 0.1 stat. sig. level without multiplicity correction

(n = 114)

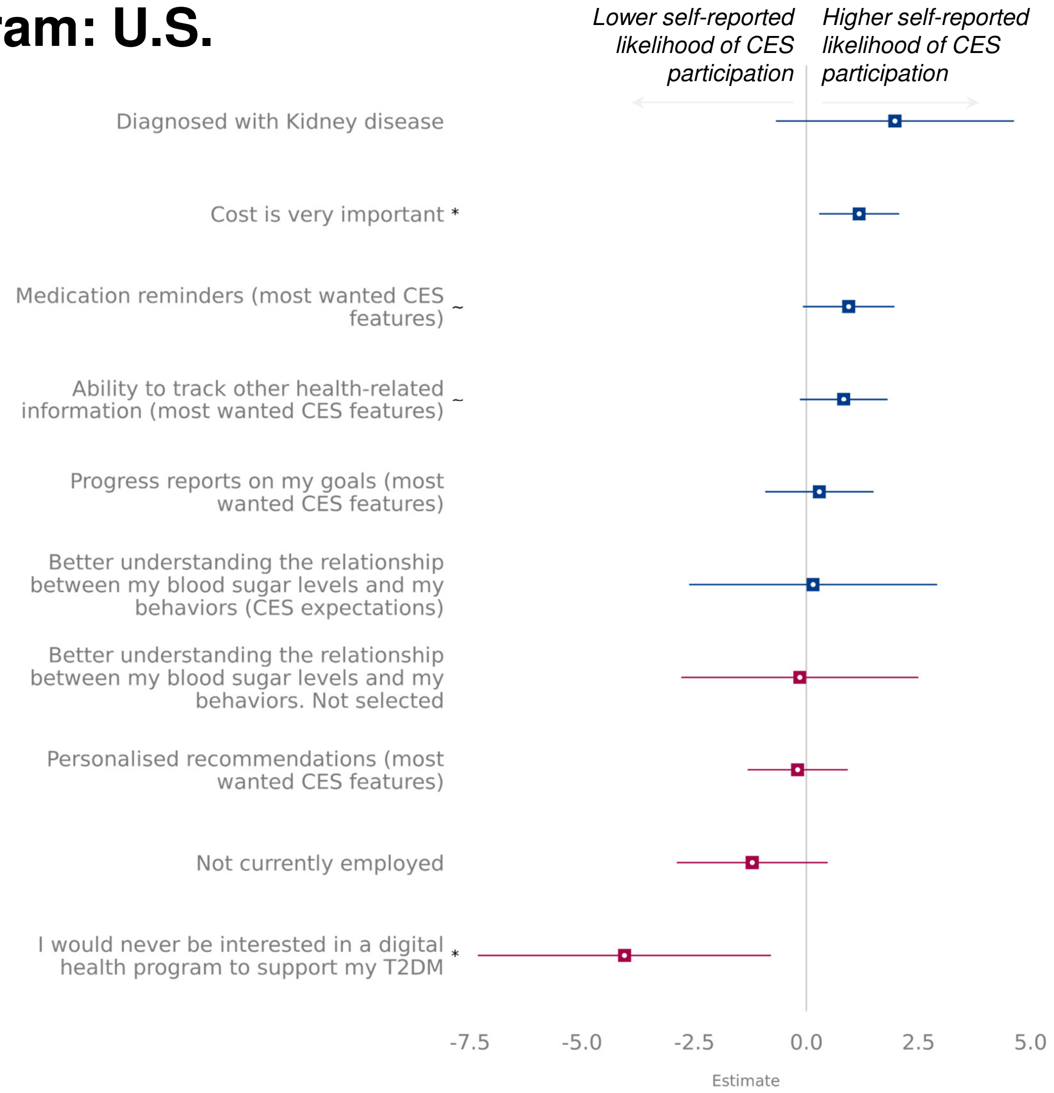

© EVIDATION HEALTH, INC. 2022

Figure S11. Factors affecting whether to participate in a CES program: Germany

The **likelihood of CES participation in Germany** tended to increase with:

- Wanting the CES to help with their **understanding of the relationship between blood sugar levels and behaviors***

and decreased along with:

- Use of **intermediate acting insulin***

* at 0.1 stat. sig. level without multiplicity correction

(n = 44) Small sample sizes limited the predictive power of the model among German participants.

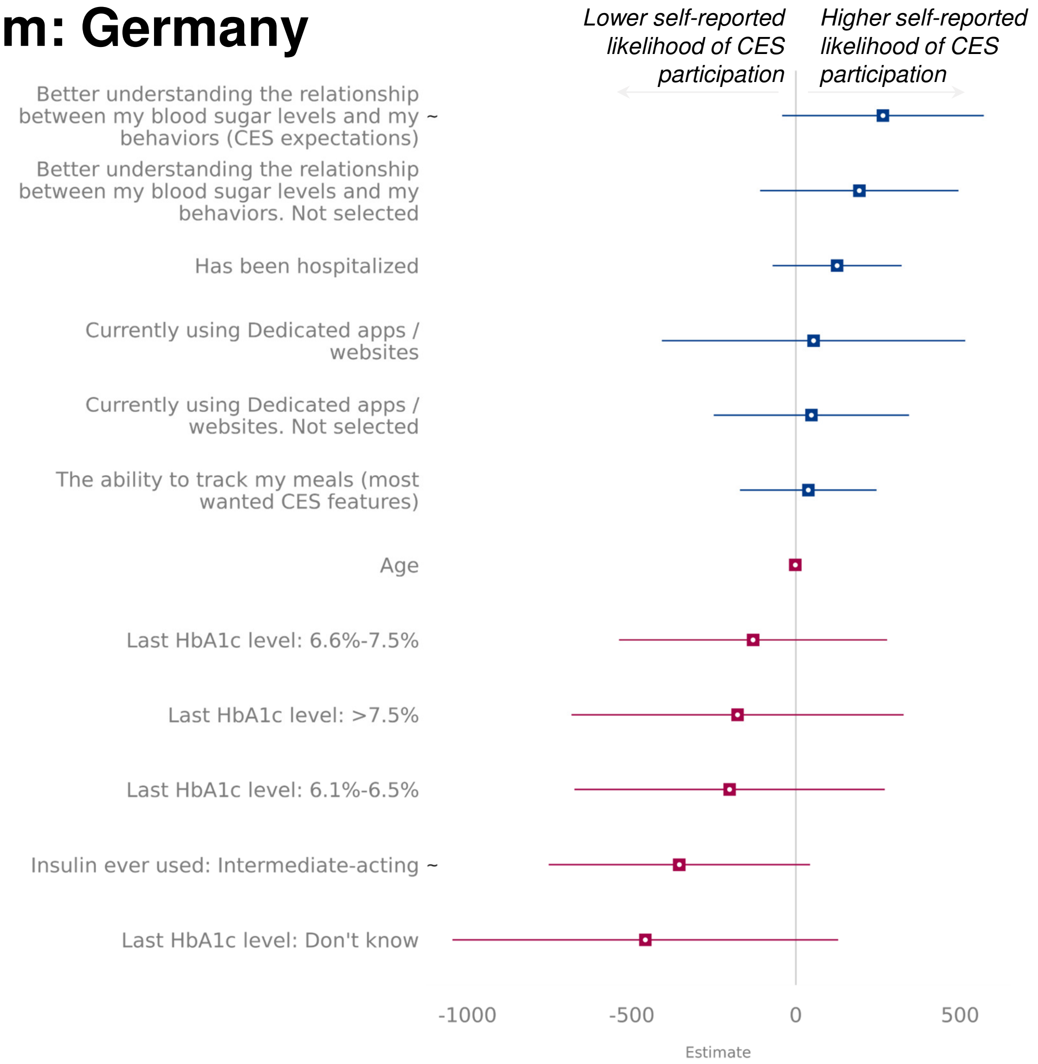

© EVIDATION HEALTH, INC. 2020

Figure S12. Factors affecting whether to participate in a CES program: France

The **likelihood of CES participation among French participants** tended to decrease with:

- Measuring blood sugar levels once per week*

* at 0.1 stat. sig. level without multiplicity correction

(n = 39) Small sample sizes limited the predictive power of the model among French participants

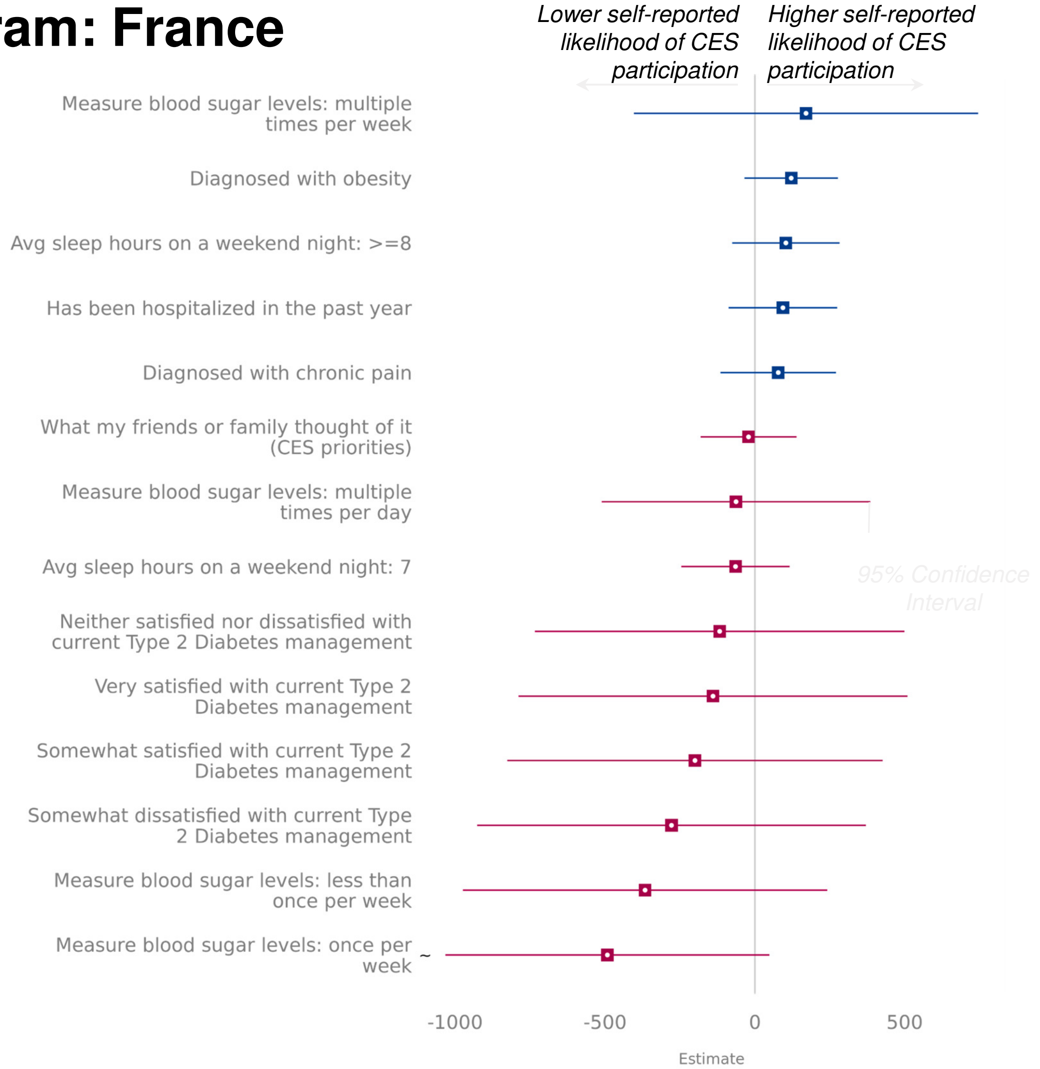

© EVIDATION HEALTH, INC. 2022
