# Supplementary material for: Patient and Physician Perspectives on the Use of a Connected Ecosystem for Diabetes Management: International Cross-Sectional Observational Study
Source: JMIR Form Res. 2023 Nov 30;7:e47145. doi: 10.2196/47145 (PMC10722356; doi:10.2196/47145)

Figure S1. Physician Attitudes towards a Connected Ecosystem

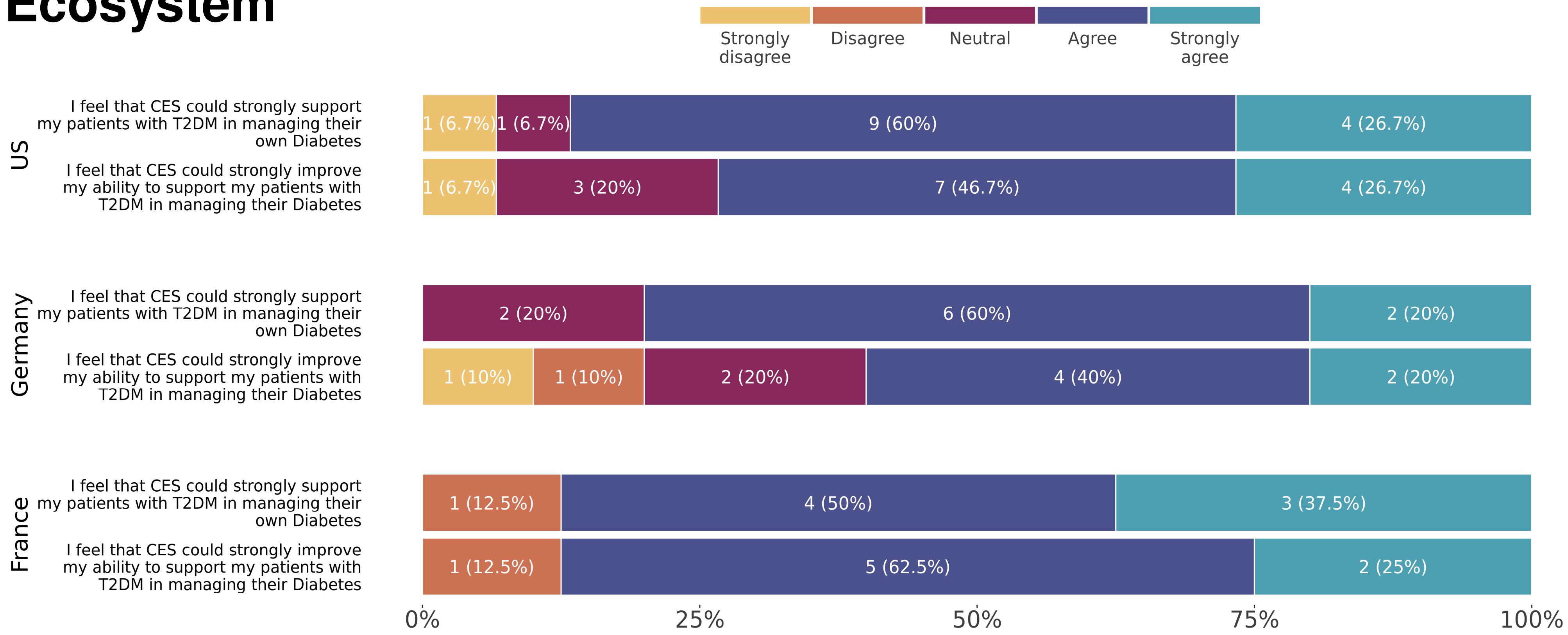

Most physicians agreed* CES could support them and their patients in managing their T2DM

* Defined as a score of 4 (Agree) or 5 (Strongly Agree) on a 5 point scale.

Figure S2. Physician Interest towards a Connected Ecosystem

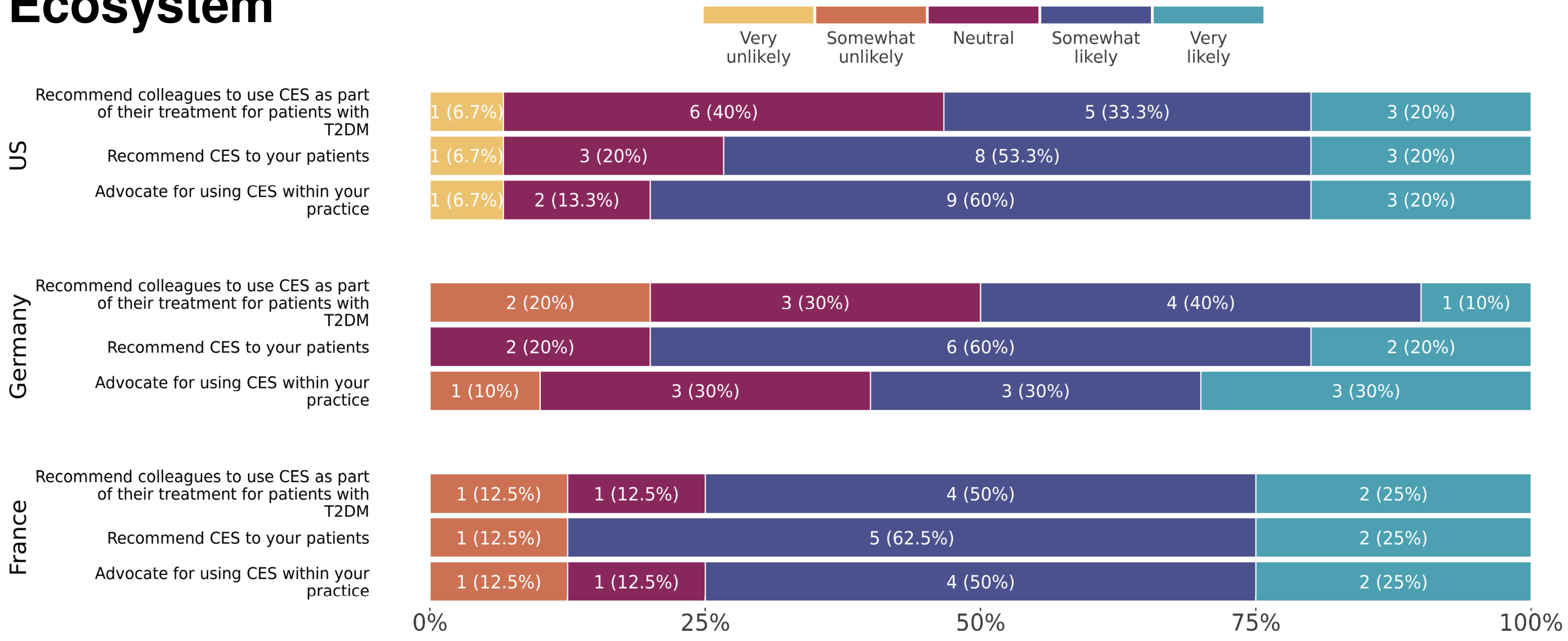

Most physicians are somewhat likely or very likely to advocate for using CES within their practice, recommend CES to their patients , and recommend CES to their colleagues

Figure S3. Connected Ecosystem Features of Interest to Physicians

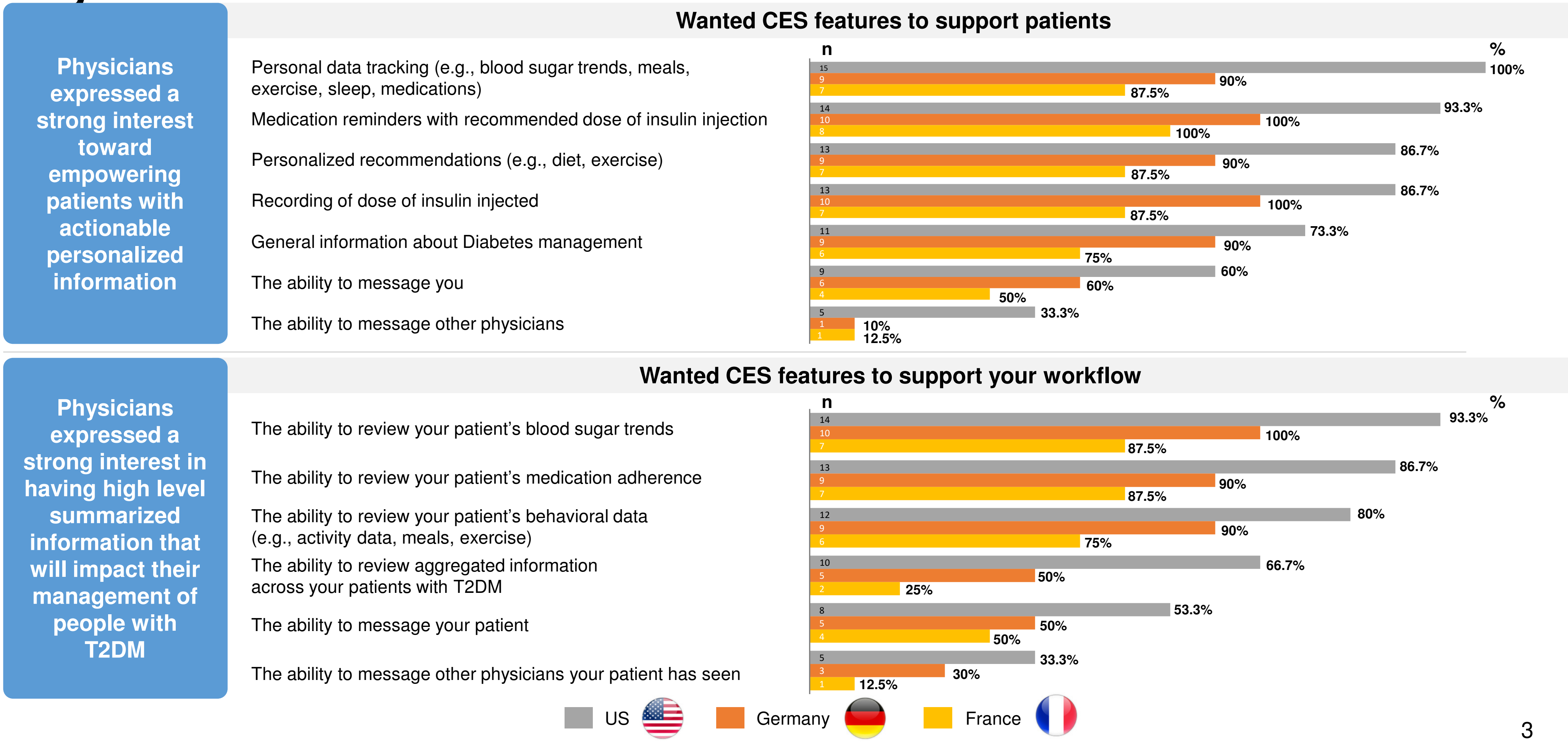

Supplement: Multimedia Appendix 3 [file formative_v7i1e47145_app3.pdf]
